# A New Three-Component Photo-Initiating System for Visible Light Recording of Volume Holograms with Single-Pulsed Laser

**DOI:** 10.3390/polym13203517

**Published:** 2021-10-13

**Authors:** Horst Berneth, Friedrich Karl Bruder, Thomas Fäcke, Sven Hansen, Koichi Kawamura, Lena Pitzer, Stephan Kern, Brita Wewer, Thomas Rölle

**Affiliations:** Covestro Deutschland AG, 51365 Leverkusen, Germany; horst.berneth@covestro.com (H.B.); friedrich-karl.bruder@covestro.com (F.K.B.); thomas.faecke@covestro.com (T.F.); sven.hansen@covestro.com (S.H.); koichi.kawamura@covestro.com (K.K.); lena.pitzer@covestro.com (L.P.); stephan.kern@covestro.com (S.K.); brita.wewer@covestro.com (B.W.)

**Keywords:** photo polymerization, radical polymerization, pulsed laser holographic recording

## Abstract

Versatile substituted electron-deficient trichloromethylarenes can easily be synthesized and combined with a Safranine O/triarylalkylborate salt to form a highly efficient three-component photo-initiation system that starts free radical polymerization to finally form holographic gratings with a single-pulsed laser. The mechanism of this photo-initiation most likely relies on an electron transfer from the borate salt into the semi-occupied HOMO of the excited dye molecule Safranine O, which after fragmentation generates an initiating alkyl radical and longer-lived dye radical species. This dye radical is most probably oxidized by the newly introduced trichloromethylarene derivative as an electron acceptor. The two generated radicals from one absorbed photon initiate the photopolymerization and form index gratings in a suitable holographic recording material. This process is purely photonic and does not require further non-photonic post treatments.

## 1. Introduction

Radiation curable materials have gained significant attention from academic groups as well as from industrial researchers over the last half century [1,2]. Several concepts have been investigated and realized covering the spectral range from extreme UV to the infrared. Because of the unique properties of diffractive optical elements, a material sensitized to visible lights is required in volume holography [3]. Here interference patterns are recorded in a photopolymer film with grating distances of approximately 100–1000 nm [4].

The origins of material developments trace back into the 1960s. Silver halide and dichromated gelatine films were used and proved to be valuable for Gabor’s three dimensional recordings of holographic images. Today’s interest is focused on holographic optical elements and their use in compact optical applications. Besides, other areas of interest are the card security and brand protection markets.

**Figure 1:**
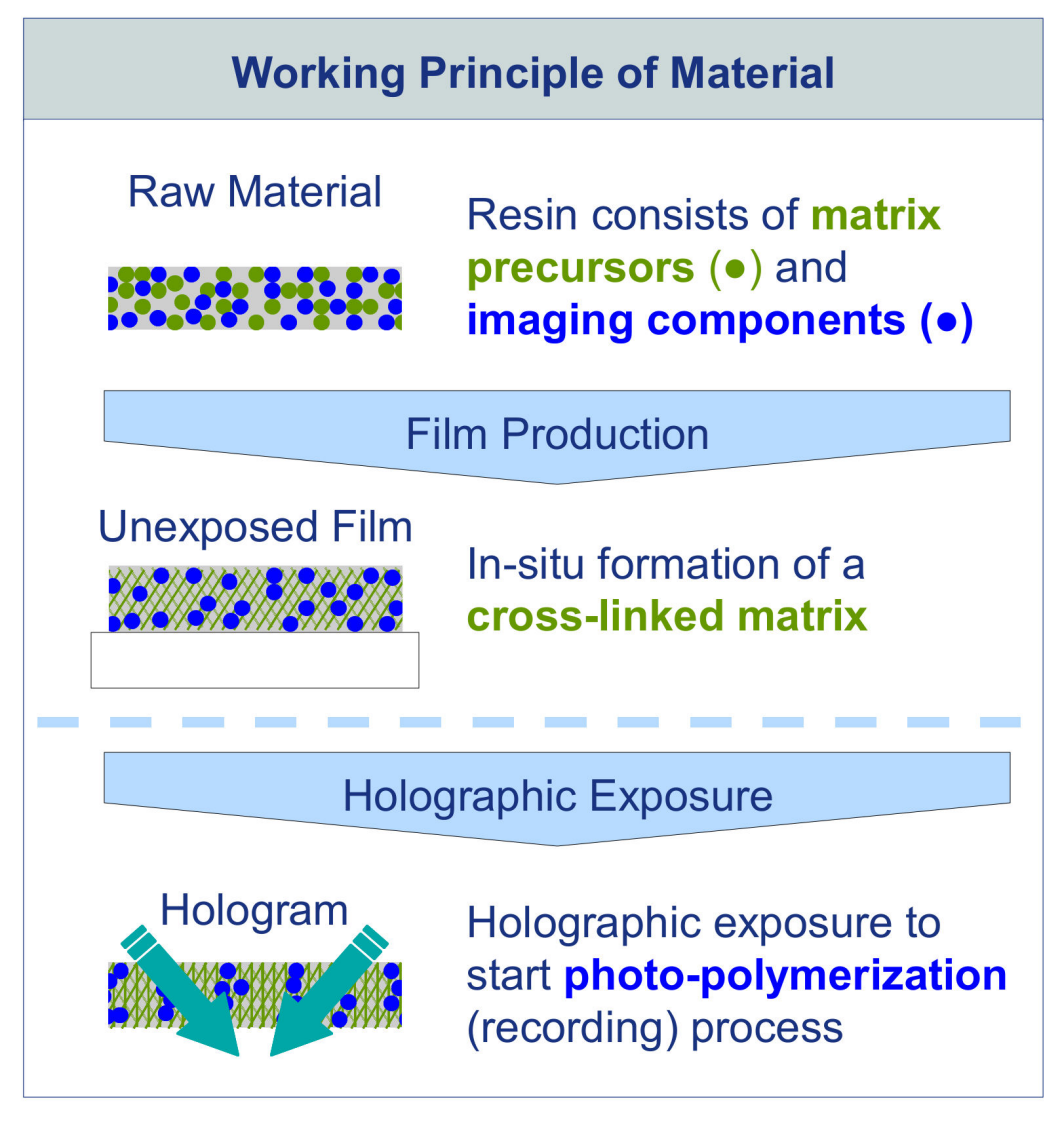
Working principle of Bayfol^®^ HX film photopolymer: the raw material is a resin that contains matrix precursors and imaging components. During film production, the matrix precursors are cross-linked to form a stable matrix system hosting the imaging components. This film product is shelf-stable until being exposed to visible light. During the recording process the film is exposed to interference pattern of two coherent laser beams to initiate the photopolymerization creating the holographic structure.

As shown in Figure 1, the photopolymer material is composed of two chemistries that do not react with each other, also so called “orthogonal chemistries” [4,5,6]. The first chemistry is composed of the matrix precursors, which are cross-linked thermally during the manufacturing process in a polyaddition reaction. This polymer network assures the mechanical stability of the recording film. The second chemistry is composed of imaging components, which are responsible for the hologram formation during light exposure. Those imaging components are triggered by light and comprise three functions: light absorption, photoinitiation, and polymerization. For light absorption a sensitizer is used that reacts with the photoinitiators forming a polymerizing radical. Subsequently, writing monomers polymerize and form the diffractive structure. 

In order to allow for a good overall performance, a perfect interplay of the mentioned major components—matrix, photo-initiating system (PIS), and writing monomers—is crucial. For example, a first requirement of this interplay is that the unexposed photopolymer, embedded in the fully cured matrix, can be stored, shipped, and handled easily under safe light conditions to prevent undesired dark photopolymerization. 

Next, the composition needs to enable photopolymerization as soon as exposed to an appropriate light source. The first step of all recording is the interaction of the light with the photo-initiator. The design of the photo-initiator is key for its spectral sensitivity, recording efficiency, and versatility as well as transparency post recording as a requirement in optical applications [7,8]. The latter is necessary to ensure high transparency and low haze in the final product.

**Scheme 1:**
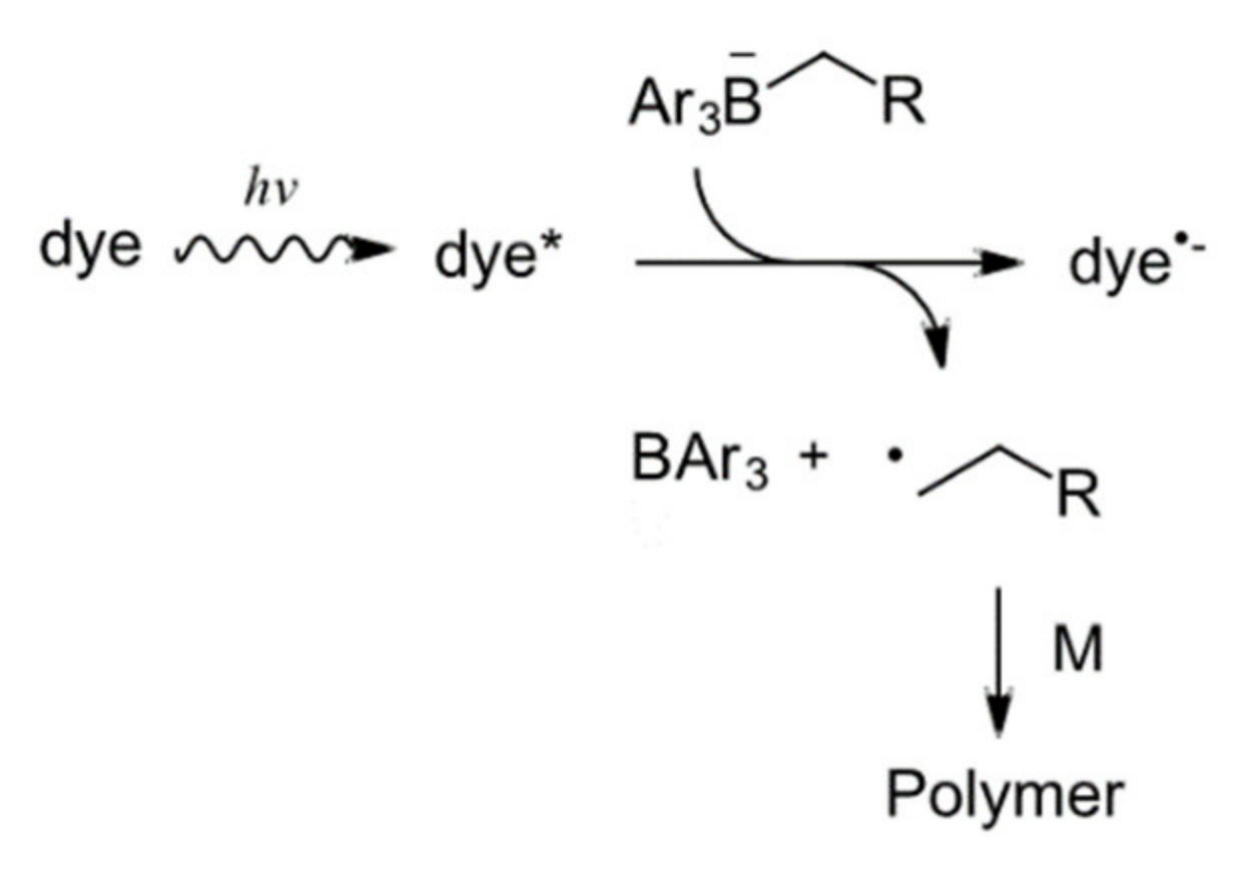
Photon-to-initiator process for a dye-triarylalkyl borate PIS.

Two significant different approaches in reference to the design of the photo-initiating systems for holographic, industrial photopolymers have been commercialized. DuPont introduced the HABI class, and Covestro (formerly Bayer MaterialScience AG) uses dye—triaryl alkyl borate salt systems (Scheme 1)—both triggering a free radical photopolymerization process. Several other PIS for free radical polymerization have been described as well: dye amine, dye onium salt, and dye triazine and single molecule photo-initiators.

The most significant requirement of the material composition is that it enables a spatially distributed photopolymerization to occur. Since the polymerizing monomers are characterized by a much higher refractive index than the matrix, a difference in refractive indices is generated between irradiated and non-irradiated areas. This spatial distribution arises from bright fringes of the interference pattern of two or more light beams that locally initiate the photopolymerization. This refractive index change is further enlarged by the writing monomers diffusion within the matrix until they react with an activated monomer or polymer chain. This process of polymerization in the bright regions and net diffusion from the dark regions causes a refractive index modulation in the material that represents the hologram. No further processing is needed to complete the hologram formation. Usually, a bleach process is performed with incoherent light to bleach the absorbing species and gain optimal transparency. This process also ensures that the writing chemistry has been fully consumed.

For recording with continuous wave lasers, this photopolymerization process described above works very efficiently. However, single-pulsed lasers as a light source are requested repeatedly to reduce the dependency of vibrations during recordings. Here, the additional challenge is to overcome problems resulting from the totally different nature of the recording process. While in conventional, continuous wave holographic recordings, the irradiating light constantly generates initiating radicals to overcome the oxygen inhibition, this is much more challenging using a single laser pulse. If a hologram should be recorded applying a laser pulse with a duration of less than a microsecond, all sensitizing molecules become excited simultaneously. Following this very efficient excitation, recombination of the exited species as well as lack of enough formed polymerizing radicals pose a challenge for sufficient writing monomer conversion.

**Scheme 2:**
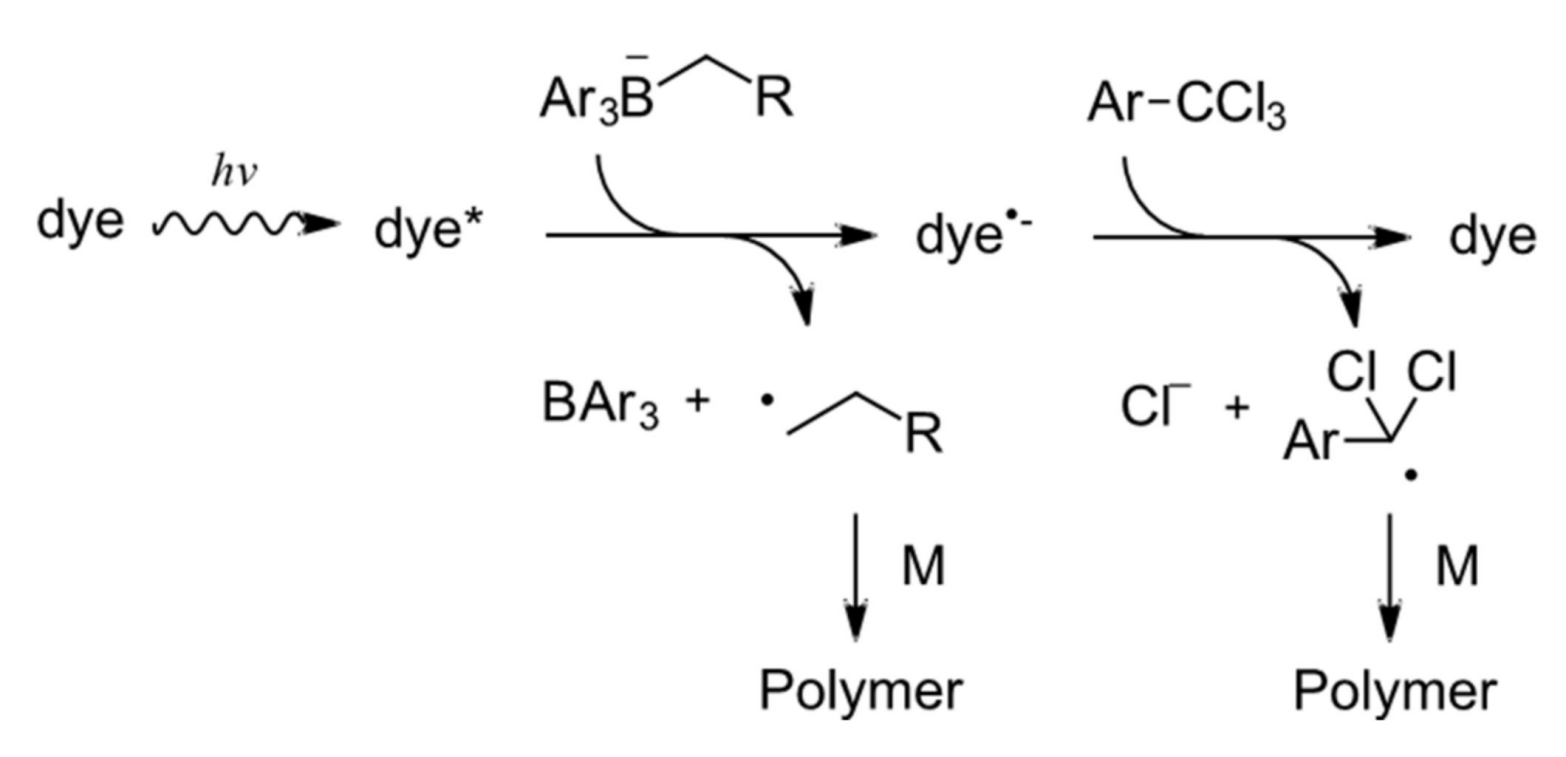
Photon-to-initiators process for a dye-triarylalkyl borate-trichloromethylaryl PIS (M = writing monomer).

In this paper we report on a new highly efficient PIS, which consists of three components [9,10]. It comprises a sensitizer, an electron acceptor, and an electron donor. Two reaction pathways become principally available depending on the nature of the sensitizer. In turn, the sensitizer (dye) governs either an oxidative or a reductive electron transfer pathway. The reductive pathway has been developed by a cationic dye, a borate salt, and a trichloromethyl substituted triazine [9,10]. Subsequently to the sensitizer (dye) photon absorption and reduction to the dye radical anion by the borate salt, the triazine oxidizes the dye radical anion and converts it back to its ground state while generating a second initiating radical by fragmentation to a chloride anion and a dichloromethine radical. This three component PIS generates up to two initiating radicals from one photon absorption (see Scheme 2). 

With our interest in a photopolymer material that is suitable for single-pulsed recording, we became interested in applying the three-component PIS for holographic recording. Moreover, we intended to expand the chemical space of the three-component PIS by exploring new oxidative additives [11]. The underlying nature of triazines as photo-initiators originates from the electron deficiency of the hetero aromatic ring, which allows for the acceptance of an electron from a donor, mostly a dye species, and the possibility to form a stabilized benzylic radical after fragmentation of a chloride ion [12]. Although partly commercially available, triazines have certain disadvantages especially regarding their poor solubility and high molecular weight.

**Scheme 3:**
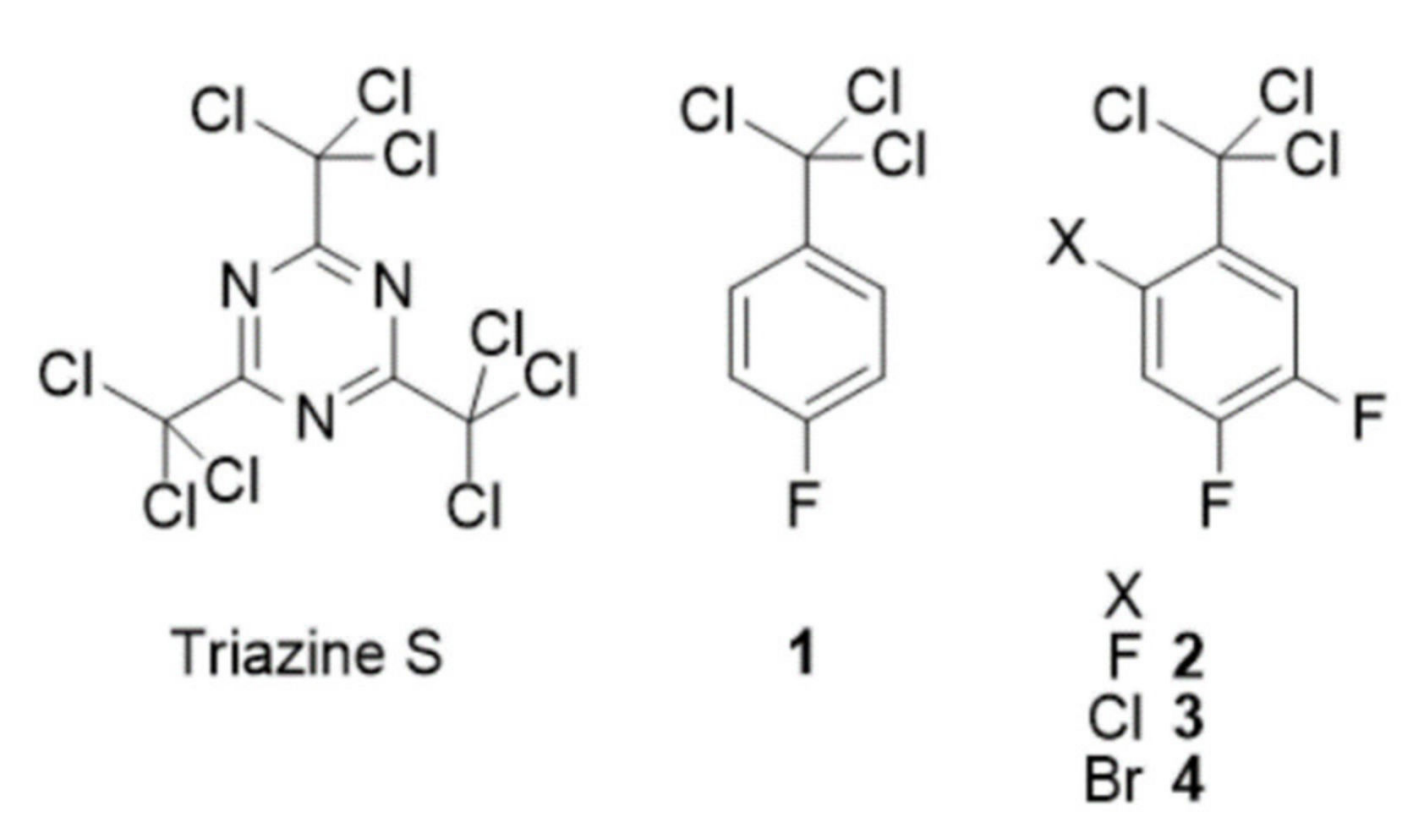
Electron acceptors investigated in this study.

We herein present an alternative approach by using electron-deficient tri-chloromethyl substituted arenes that proved their capabilities to be used in photopolymers for single-pulsed holographic recording (Scheme 3).

These molecules were identified as potential candidates in a computational study, which was then proven experimentally.

## 2. Materials and Methods

The investigated photo polymers consist of three major constituents. The first part is a three dimensionally cross-linked aliphatic polyurethane formed from commercially available precursors. A difunctional A-B-A block co-polyester polyol with a mean molecular weight of 2000 g/mol and a hydroxyl value of 54–58 mg KOH/g is catalytically reacted with the low viscous aliphatic polyisocyanate cross linker Desmodur^®^ N 3900 with an average NCO content of 23.5% and a viscosity of ~730 mPas at 23 °C, both available from Covestro Deutschland AG, Leverkusen, Germany. 

**Scheme 4:**
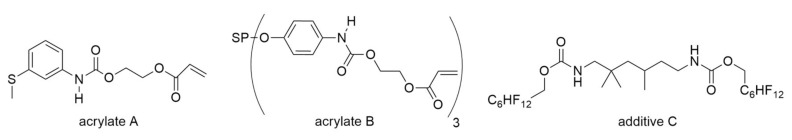
Photopolymer components used in this study.

The second part contains the writing chemistry that during the holographic exposure forms the refractive index gratings. Here, a mixture of the monofunctional acrylate A (2-(([3-(methylsulfanyl)phenyl]carbamoyl}oxy)-ethylprop-2-enoate), the trifunctional acrylate B ((phosphorothioyltris(oxybenzol-4,1-diylcarbamoyloxyethan-2,1-diyl) tris acrylate), and the additive C bis(2,2,3,3,4,4,5,5,6,6,7,7-dodecafluorheptyl)-(2,2,4-trimethylhexan-1,6-diyl) bis carbamate is utilized at the fixed weight ratio of 20:20:15 for all described experiments, see Scheme 4 [13]. 

**Scheme 5:**
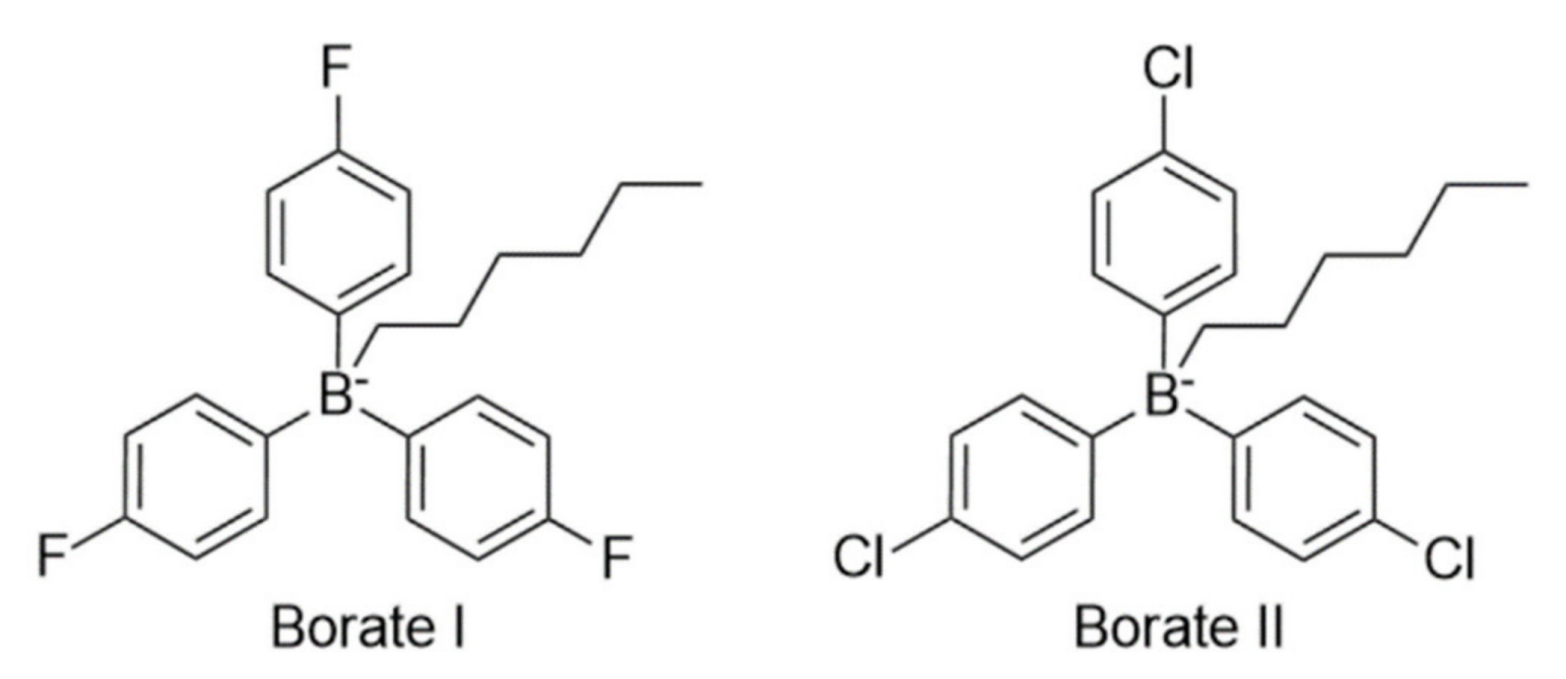
Electron donors investigated in this study (counter ion is tetra-butyl ammonium).

The third part consists of one cationic dye, preferably Safranine O, an alkyl triaryl borate salt as electron donor component, and a more closely described electron acceptor component. The electron-donating properties of the alkyl triaryl borate salts can be nicely adopted by the substitution pattern of the aryl moieties and the nature of the generated primary radical modified by the selection of the alkyl group. We selected for this study para-fluoro and para-chloro substituted borates that have a reduced reduction potential compared to the unsubstituted triphenylalkyl borates. As the alkyl chain, hexyl was chosen. The two hexyltriarylborate tetra-butyl ammonium salts I and II were prepared according to published procedures and used herein, *cf.* Scheme 5 [14,15,16].

The electron acceptors **2**–**4** had to be synthesized from the respective commercially available precursors by an Friedel-Crafts alkylation using carbon tetrachloride as highly efficient electrophilic reagent [17]. The products were obtained as crystalline solids in good-to-excellent yields.

**Figure 2:**
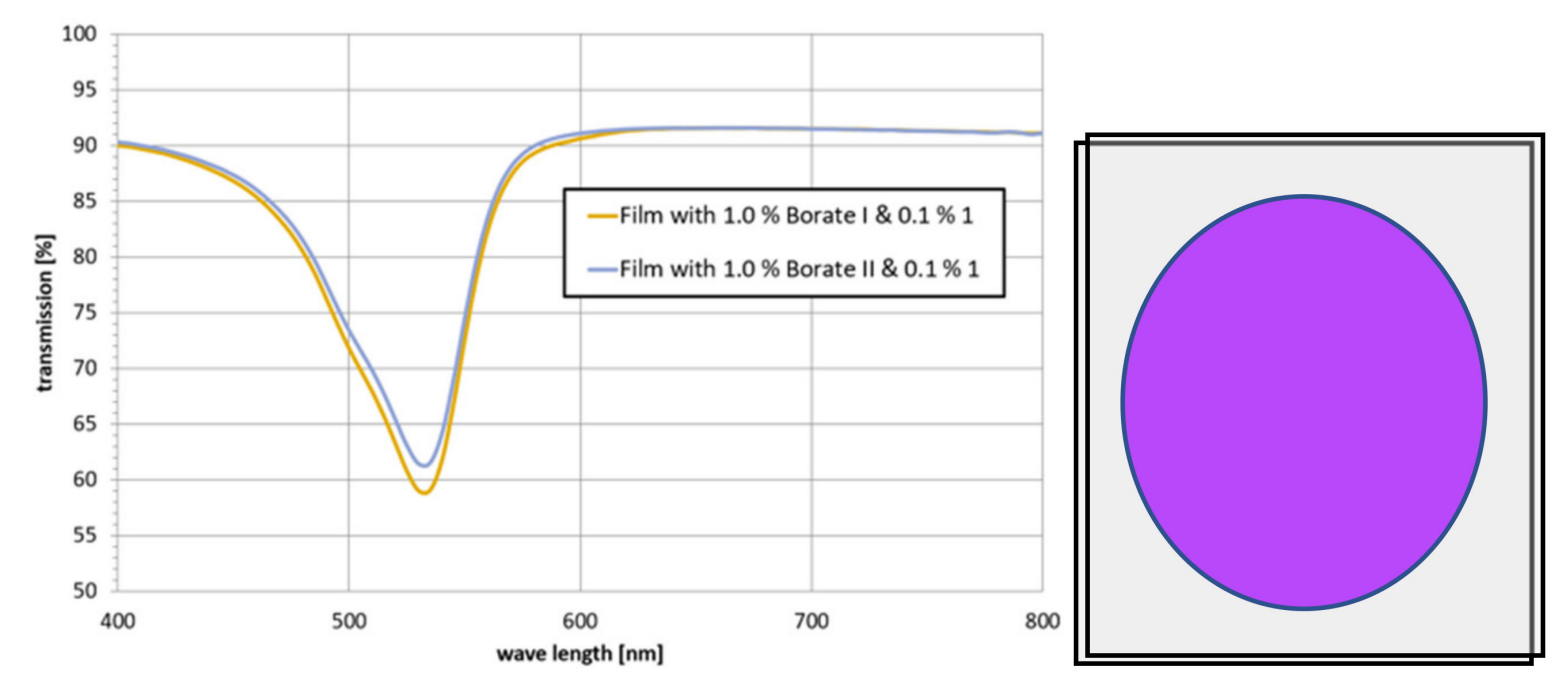
Left—VIS transmission spectra of the two photopolymer film media with 1.0% of either borate I or II in combination with 0.1% of 4-Fluorotrichlorobenzene **1**; right—respective drawing of a film.

Ultimately, in order to prepare recordable films, all ingredients had to be checked for their compatibility and stability under the conditions. One very important aspect is the homogeneity and planarity of the photopolymer film. In order to successfully obtain the latter, all ingredients are thoroughly mixed in the dark. The open time processing window for a pre-mixture herein is in the order of 60 min, and the viscous liquid is applied between two optical planar glass plates and cured at 25 °C for 24 h. This typically yields photopolymer films of ~40 × 40 mm in size at a layer height of 15–20 µm, Figure 2.

These film samples are now ready for holographic recording. It is very important—in particular for large-scale industrial production of holographic media—that the photosensitivity is sufficient to achieve large-area exposure with any given source of laser light without loss of index modulation. Particularly the choice of a suitable photo-initiator here is of decisive importance for the properties of the photopolymer. 

However, holographic exposure using a continuous source of laser light comes up against technical limits in the case of large-area exposure, since efficient formation of the hologram will always require a certain minimum of light per unit area, and the technically available laser power is limited. Large-area exposures at a comparatively low dosage of radiation additionally require long exposure times, which in turn impose very high requirements on the mechanical damping of the exposure set-up to eliminate vibration.

A further possible way to achieve large-area exposure of holograms consists in using very short pulses of light, for example from pulsed lasers or continuous wave lasers in conjunction with very fast shutters. Pulse durations with pulsed lasers are typically 500 ns or less. Pulse durations with continuous wave lasers and very fast shutters are typically 100 µs or less. In effect, the same amount of energy can be introduced here as with continuous lasers in seconds. Holograms can be optically printed dot by dot as known from conventional printing techniques. Since pulsed lasers or fast optical shutters are technically available, and an exposure set-up of this type has very low requirements with regard to mechanical damping to eliminate vibration, this represents a good technical alternative of the above-described set-ups involving continuous lasers for large-area exposure of holograms.

To determine the diffraction efficiency in pulsed exposure, a Denisyuk hologram of a mirror was recorded in a sample consisting of two glass plates included with a photopolymer film. The sample was exposed with its planar face perpendicular to the laser beam. The distance between the sample and the mirror was 3 cm.

**Figure 3:**
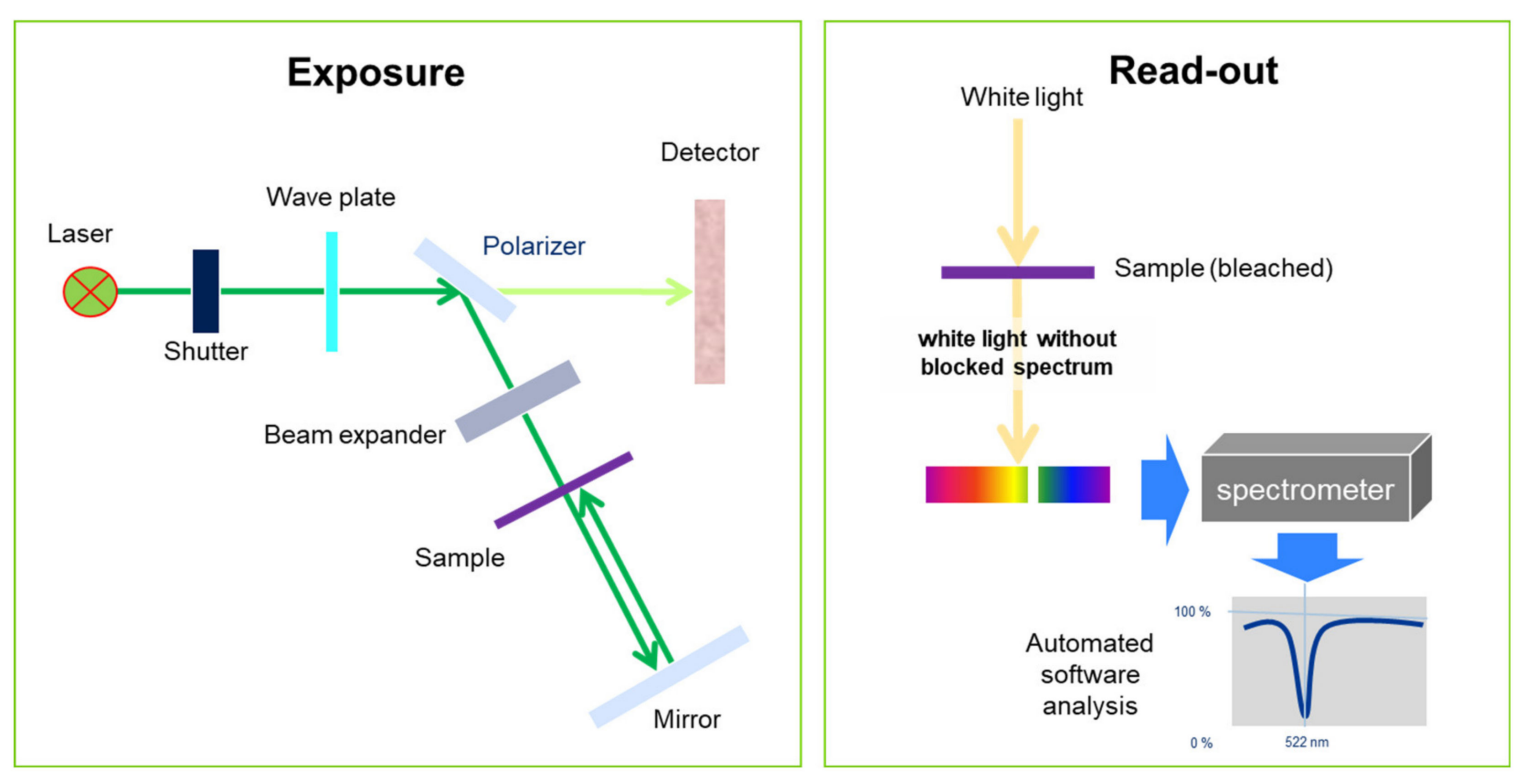
Schematic representation of the pulsed laser recording and read-out setup.

As the laser, a Brilliant b pulsed laser from Quantel (Lannion, France) was used. The laser was equipped with a Q-switched Nd-YAG laser frequency doubled to 532 nm. The single frequency mode was guaranteed by a seed laser. Coherence length was arithmetically about 1 m. Pulse duration was 4 ns, and average power output was 3 W at a pulse repetition rate of 10 Hz, Figure 3.

The electronically controlled shutter was used to ensure a single-pulse exposure. The waveplate rotates the polarization plane of the laser light, and the subsequent polarizer was used to reflect the S-polarized portion of the laser light in the direction of the sample. The exposed area was adjusted by beam expansion. The waveplate and the beam expander were adjusted such that the sample was given an exposure dosage of 100 mJ/cm^2^/pulse.

To determine the diffraction efficiency, the samples were each exposed with exactly one pulse. After exposure, the sample was bleached on a light table.

A transmission spectrum was measured through the hologram of the bleached sample using an HR4000 spectrometer from Ocean Optics (Ostfildern, Germany). The sample was placed perpendicularly to the light beam. The transmission spectrum showed a transmission collapse at a wavelength at which the Bragg condition was satisfied. The UV spectrum was automatically evaluated after baseline adjustment, and peak_rise_ (height of the peak at the reconstruction maximum from the baseline) and FWHM were determined. This transmission collapse to the baseline was evaluated as the diffraction efficiency DE of the Denisyuk hologram of the mirror. Index modulation Δn according to Kogelnik was calculated [18]. 

Computation of the electron acceptor properties and, in turn, the reduction potential of the triazines A and S and the electron-deficient trichloromethyl arenes was carried out with GAMESS on a suitable personal computer [19,20]. All computations were executed in a PCM solvent model with acetonitrile as the solvent [21]. Geometry optimizations were carried out, and the electronic ground state was thus determined and confirmed by a frequency calculation. In order to reflect the molecular processes, one chloride ion of one trichloromethyl group was removed from the minimized and confirmed ground state geometry, and one electron was added. This new geometry was fixed, and the electronic energy was calculated (now UHF). All obtained geometries and frequency run results are listed in the Supplementary Material. The reduction potential was subsequently calculated using Nicewicz’s protocol, and the derived data yielded the corresponding reduction potential values against the standard calomel electrode [22].

## 3. Results

### 3.1. Electronic Comparison of Electron-Deficient Benzenes with Triazines S and A

**Scheme 6:**
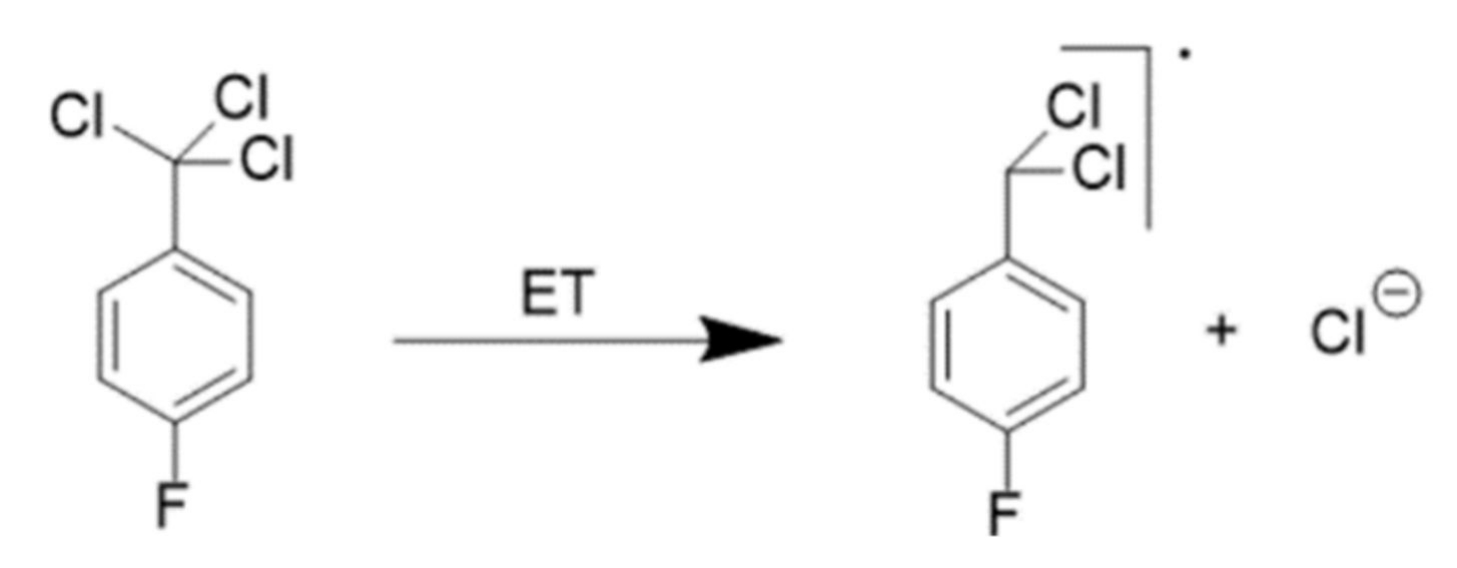
Isodesmic reaction scheme after electron transfer (ET) exemplified with 4-fluorotrichlorobenzene (**1**).

Trichloromethyl-triazines are well established components of radical photo-initiators and thus have been electronically well investigated and described. Herein, we report a straightforward method for the comparison of electron acceptors. We slightly adopted the proposal by Nicewicz for dissociating systems [22]. Geometry-optimized ground states of the investigated molecules were determined as a local minimum on the potential hypersurface and in the next step oxidized in silico, i.e., one chloride anion was dissociated from that geometry to yield the final stage after electron transfer; this is schematically displayed in Scheme 6 with 4-fluorotrichlorobenzene (**1**) as example. That approach reflects a purely thermodynamic picture, but given the fast dynamics in the overall involved electronic processes, movements of the core atoms are fully and consequently neglected during computation as described first by Born and Oppenheimer [23]. 

B3LYPV1R-KTZVPP energy calculation and consecutive data treatment following Nicewicz’s protocol for triazine S, triazine A, 4-fluorotrichlorobenzene (**1**), 1,2,4-trifluoro-5-(trichloromethyl) benzene (**2**), and 1-chloro-4,5-difluoro-2-(trichloromethyl)benzene (**3**), all yielded very similar reduction potentials of −1.10 ± 0.05 V vs. SCE, respectively [22]. The deviation of the result obtained with B3LYPV1R-6-31+G(d,p) for 1-bromo-4,5-difluoro-2-(trichloromethyl)benzene (**4**) (−0.84 V) can be explained by the lower-level basis employed herein. The reported values are in good agreement, since there is only a minor shift of ~0.10 V compared with the experimentally reported reduction potential for triazine A (TA) and triazine S (TS) (−1.00 V vs. standard SCE) [24]. The results are compiled in Table 1.

### 3.2. Radical Photopolymerization Initiated by Safranine O/Triarylalkylborate Salt/Trichloromethyl Arenes

Recording of holograms with pulsed laser light in the visible range requires a photopolymer with a significantly improved sensitivity and efficiency compared to currently available materials. Three-component PIS have been widely considered as more efficient than comparable two-component systems. The two para-fluoro/para-chloro substituted borate salts I and II were combined with triazine S, 4-fluorotrichlorobenzene (**1**), 1,2,4-trifluoro-5-(trichloromethyl) benzene (**2**), 1-chloro-4,5-difluoro-2-(trichloromethyl)benzene (**3**), and 1-bromo-4,5-difluoro-2-(trichloromethyl)benzene (**4**), respectively. The formed photo polymer of 10–15 μm thickness was consecutively irradiated with a single 532 nm laser pulse of 100 mJ/cm^2^, and after bleaching, the performance was evaluated by the observed transmission in the visible spectrum.

This spectral response is reported as peak_rise_, full width at half maximum (FWHM), and the index modulation Δn. The higher the Δn number, the higher the efficiency of the holographic function, a mirror function in our case, and this in turn translates into a higher efficient photo-initiation process. Holograms cannot be recorded with photo polymers containing 0.1% or 1.0% triazine S with either borate I or borate II, although the efficiency of those three-component PIS is well documented. In contrary, all photo polymer films containing the tri-chloromethyl substituted arenes are well suited for single-pulse holographic recording. 

**Figure 4:**
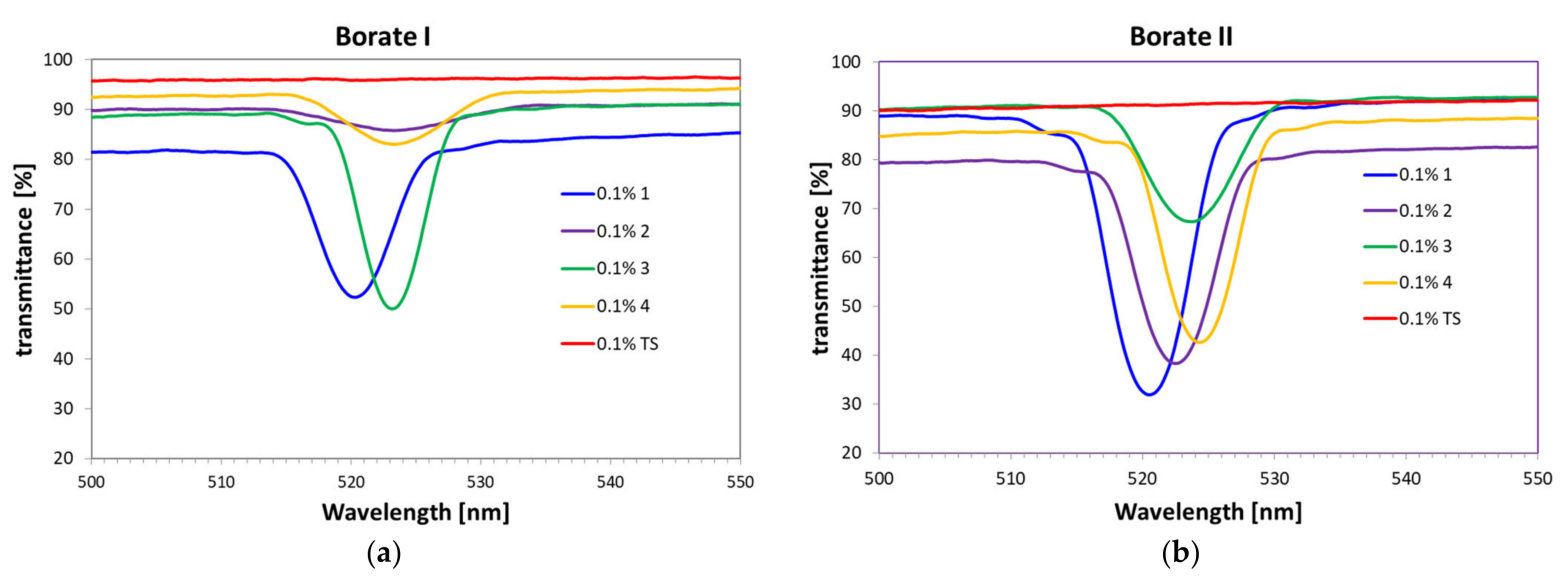
(**a**) Transmittance spectra of photopolymer films after single-pulse recording containing borate I (**a**) and borate II (**b**).

The spectral results for peak_rise_, FWHM, and Δn are compiled in Table 1, and the transmission spectra for photo polymer films with each 0.1% electron acceptor and borate I are plotted in Figure 4a and analogously for borate II in Figure 4b.

## 4. Discussion

As can be seen in Table 1 and in the two transmission spectra in Figure 4a,b, the different PIS have completely different outcomes in terms of holographic performance. At first, a two-component PIS was tested using only the well-known cationic dye Safranine O and either one of the borate salts I or II. In this case, no hologram could be written with a single laser pulse (see entry 1 in Table 1). This indicates that a two-component PIS is not efficient enough for such challenging recording conditions. Each absorbed photon generates at best one initiating radical from the fragmentation of the triarylalkylborate salt, which might not be enough to overcome the oxygen inhibition and start the polymerization process. Next, a three-component PIS was utilized comprising Safranine O, either one of the borate salts I or II, and the established and commercially available co-initiators triazine A or triazine S. Even though these photopolymers contained a three-component PIS, no holograms could be recorded (see entries 2–4 in Table 1 and red line in the transmission spectra in Figure 4a,b). Most probably, the added triazines did not work as electron acceptors in these cases, which might be due to their poor solubility in the photopolymer. Additionally, the very low reduction potential of triazine S could also be a reason for this failure, as it might be too low in order to enable an exothermic electron transfer with the reduced Safranine O species. At this stage, the novel tri-chloromethyl substituted arene derivatives were employed as co-initiators in single-pulse holographic recordings. All four tested co-initiators led to hologram recordings with both borates I and II (see entries 5–8 in Table 1). The photopolymers containing borate salt II as a first co-initiator worked on average slightly better than the ones containing borate salt I, as can be deduced when comparing the Δn-values of the recorded holograms. The combination of Safranine O, borate salt II, and electron acceptor 1 gave in total the best performance, with a Δn-value greater than 0.01 and a peak rise of 59% for this single-pulse recording. Regarding the various tri-chloromethyl substituted arene derivatives, it is difficult to evaluate which one works best in general. For borate salt I, the electron acceptor **3** works best, whereas for borate salt II, electron acceptor **1** ranks highest. As their reduction potentials are almost the same, the differences in holographic performance might be due to slightly different solubilities or weak intermolecular forces like π-π stacking with the reduced Safranine O species that promote the crucial electron transfer. In general, the fact that all of the novel trichlormethyl substituted arenes worked well in these single-pulse holographic recordings supports the proposed mechanism depicted in Scheme 2. The second initiating radical generated by the fragmentation of the reduced trichloromethyl moiety might be essential for the initiating process, as it doubles the amount of initiating radicals generated per photon. The calculated reduction potentials of these electron acceptors further support this mechanistic picture. 

## 5. Conclusions

It was shown that versatile substituted electron-deficient trichloromethyl arenes can easily be synthesized and combined with a Safranine O/triarylalkylborate salt to form highly efficient three-component photo-initiation systems that start free radical polymerization and record holographic gratings with a single laser pulse. The proposed mechanism of this photo-initiation comprises an electron transfer from the borate salt into the semi-occupied HOMO of the excited dye molecule Safranine O, which generates, after fragmentation, an initiating alkyl radical and longer lived dye radical species. This dye radical is subsequently oxidized by the newly introduced trichloromethylarene derivative as an electron acceptor. The fragmentation of the trichloromethylaryl radical anion to a chloride and a dichlorobenzyl radical finalizes the photo-initiation reaction cascade. Converting one absorbed photon into two radicals capable to initiate a radical polymerization is seen as the major benefit of this concept. This pure photonic process does not require further non-photonic post treatments. 

## Figures and Tables

**Table 1:** Reduction potential of the electron acceptors and holographic performance of derived photo polymer films in single-pulsed holographic recording.

Electron Acceptor	Calculated Reduction Potential/V	Borate I			Borate II		
		Peak_rise_/%	FWHM	Δn	Peak_rise_/%	FWHM	Δn
none		0	0	0	0	0	0
0.1% TS	−1.15 ^1^	0	0	0	0	0	0
1.0% TS	−1.15 ^1^	0	0	0	0	0	0
TA	−1.10 ^1^	-	-	-	-	-	-
0.1% **1**	−1.10 ^1^	31	6.3	0.0072	59	6.8	0.0106
0.1% **2**	−1.09 ^1^	5	10.1	0.0047	43	6.6	0.0088
0.1% **3**	−1.07 ^1^	40	5.4	0.0070	24	7.3	0.0073
0.1% **4**	−0.84 ^2^	10	8.3	0.0054	44	6.2	0.0083

^1^ B3LYPV1R-KTZVPP; ^2^ B3LYPV1R-6-31+G(d,p).

## Data Availability

Please refer to the Associated Supplementary File.

## Supplementary Materials

The following are available online at https://www.mdpi.com/article/10.3390/polym13203517/s1.
