# Supplementary material for: A New Three-Component Photo-Initiating System for Visible Light Recording of Volume Holograms with Single-Pulsed Laser
Source: Polymers (Basel). 2021 Oct 13;13(20):3517. doi: 10.3390/polym13203517 (PMC8539560; doi:10.3390/polym13203517)
Supplement: Supplementary file 1 [file polymers-13-03517-s001.zip › polymers-1383573-supplementary.pdf]

The solvents used were obtained commercially without further purification.
Dibutyltin dilaurate [77-58-7] is available from Merck KGaA, Darmstadt.
Desmodur® N 3900 Product from Covestro Deutschland AG, Leverkusen, Germany, hexane diisocyanate-based polyisocyanate, proportion of imino-oxadiazinedione at least 30%, NCO content: 23.5%.
Fomrez UL 28 Urethanization catalyst, available from Momentive Performance Chemicals, Wilton, Conn., USA.
Aluminum chloride [7446-70-0] is available from Acros Organics, Geel, BE.
1-Chloro-3,4-difluorobenzene [696-02-6] is available from ABCR GmbH & CO. KG, Karlsruhe.
1-Bromo-3,4-difluorobenzene [348-61-8] is available from ABCR GmbH & CO. KG, Karlsruhe.
4-Chlorophenylmagnesium bromide [873-77-8] is available as 0.9 M solution in THF/toluene from Merck KGaA, Darmstadt.
4-Fluorophenylmagnesium bromide [352-13-6] is available as 1.0 M solution in THF from Merck KGaA, Darmstadt.
4-Fluorotrichlorobenzene [402-42-6] is available from ABCR GmbH & CO. KG, Karlsruhe and was used as obtained.
Tetrabutylammonium bromide [1643-19-2] is available from ABCR GmbH & CO. KG, Karlsruhe.
Safranine O [10309-89-4] is available from Chemos GmbH, Germany, Article No. 1308. The composition of the substance mixture is described in [1].
Sodium bis(2-ethylhexyl) [45297-26-5] is available from Merck KGaA, Darmstadt.

Minimized molecular geometry of 2,4,6-tris(chloromethyl)-1,3,5-triazine (TS)

C	6.0	5.5207346322	-8.9955781966	5.4292639485
N	7.0	6.3416876274	-7.9612632533	5.5714553663
C	6.0	5.9244818403	-6.8418837550	5.0107472629
N	7.0	4.7691749804	-6.6881703616	4.3777926969
C	6.0	4.0371907676	-7.7827250300	4.2911384891
N	7.0	4.3655154751	-8.9662754402	4.7944983317
C	6.0	6.8662926638	-5.6294316090	5.1228588661
CL	17.0	6.2860330863	-4.2450712419	4.1718395985
CL	17.0	8.4951507951	-6.0996579711	4.5413126331
CL	17.0	6.9596791790	-5.1647125508	6.8561310065
C	6.0	5.9717213322	-10.3115936922	6.0875656464
C	6.0	2.6871697788	-7.7193870598	3.5565314030
CL	17.0	2.7582969891	-8.8590238603	2.1700065375
CL	17.0	2.3246994795	-6.0873583747	2.9565318629
CL	17.0	1.3946054490	-8.2327628868	4.6901035273
CL	17.0	6.2174521115	-10.0076050665	7.8397855928
CL	17.0	4.7752092966	-11.6087043188	5.8841639695
CL	17.0	7.5236615162	-10.8196883314	5.3417772612

Calculated IR frequencies of 2,4,6- tris(chloromethyl)-1,3,5-triazine (TS)

MODE	FREQ(CM-1)	SYMMETRY	RED.MASS	IR-INTENS.
1	6.850	A	29.944453	0.000086
2	0.793	A	29.391869	0.000001
3	0.074	A	23.820047	0.000000
4	0.050	A	23.819319	0.000000

5	0.290	A	24.556584	0.000000
6	2.564	A	29.877735	0.000002
7	7.152	A	33.412977	0.000090
8	10.999	A	34.654833	0.000068
9	12.614	A	34.671530	0.000335
10	57.823	A	24.861152	0.004928
11	81.266	A	30.938242	0.000325
12	83.153	A	29.340102	0.000466
13	90.772	A	22.177524	0.000072
14	106.571	A	20.804885	0.000198
15	159.732	A	32.179364	0.007582
16	164.836	A	32.888354	0.007195
17	179.311	A	33.122246	0.007255
18	189.166	A	33.713271	0.005308
19	210.502	A	20.601400	0.004171
20	225.490	A	20.860317	0.000789
21	236.774	A	24.367632	0.001504
22	265.312	A	24.939346	0.003646
23	268.558	A	28.152989	0.000109
24	269.876	A	26.364158	0.000666
25	288.066	A	27.126819	0.000736
26	291.663	A	25.695036	0.000586
27	328.751	A	22.303617	0.054071
28	348.458	A	22.829705	0.109879
29	353.167	A	23.033817	0.064868
30	405.602	A	32.672393	0.001485
31	427.804	A	24.848779	0.037945
32	429.795	A	24.084217	0.007678
33	502.916	A	15.118789	0.009306
34	639.242	A	13.408439	0.001928
35	648.241	A	13.428399	0.005858
36	701.208	A	13.260187	3.164508
37	703.988	A	13.267017	3.075483
38	727.717	A	13.033935	8.792740
39	796.600	A	12.694655	3.250610
40	801.260	A	12.687398	2.257302
41	803.138	A	12.259370	0.074338
42	808.867	A	12.222358	0.017590
43	832.311	A	12.594725	0.059997
44	844.251	A	12.525594	0.207781
45	875.459	A	12.627841	0.077086
46	1018.912	A	13.889923	0.044028
47	1019.315	A	12.693657	0.427347
48	1023.720	A	12.697769	0.910919
49	1212.454	A	12.769472	0.033731
50	1362.376	A	12.019164	0.090443
51	1368.893	A	12.553092	2.727608

52	1371.580	A	12.549893	2.824464
53	1580.580	A	12.758427	7.577076
54	1583.688	A	12.759998	6.786063

Molecular geometry for energy calculation of Bis-(trichloromethyl)-dichloromethyltriazin

C	6.0	5.5207346322	-8.9955781966	5.4292639485
N	7.0	6.3416876274	-7.9612632533	5.5714553663
C	6.0	5.9244818403	-6.8418837550	5.0107472629
N	7.0	4.7691749804	-6.6881703616	4.3777926969
C	6.0	4.0371907676	-7.7827250300	4.2911384891
N	7.0	4.3655154751	-8.9662754402	4.7944983317
C	6.0	6.8662926638	-5.6294316090	5.1228588661
CL	17.0	6.2860330863	-4.2450712419	4.1718395985
CL	17.0	8.4951507951	-6.0996579711	4.5413126331
CL	17.0	6.9596791790	-5.1647125508	6.8561310065
C	6.0	5.9717213322	-10.3115936922	6.0875656464
C	6.0	2.6871697788	-7.7193870598	3.5565314030
CL	17.0	2.7582969891	-8.8590238603	2.1700065375
CL	17.0	2.3246994795	-6.0873583747	2.9565318629
CL	17.0	1.3946054490	-8.2327628868	4.6901035273
CL	17.0	6.2174521115	-10.0076050665	7.8397855928
CL	17.0	4.7752092966	-11.6087043188	5.8841639695

Minimized molecular geometry of 2-(4'-methoxyphenyl)-4,6-bis(trichloromethyl)-1,3,5-triazine (TA)

N	7.0	-4.2456334460	-1.6804640728	-0.1374739666
C	6.0	-4.1697603114	-2.9797721468	0.0334018400
N	7.0	-3.0731435436	-3.6703306392	0.3224139424
C	6.0	-1.9839841861	-2.9195933742	0.4180878415
N	7.0	-1.9295759718	-1.6133468114	0.2915154262
C	6.0	-3.0950041538	-0.9973352204	0.0221491905
C	6.0	-5.4811533698	-3.7848156211	-0.0492272805
C	6.0	-0.6711061810	-3.6434117218	0.7752851895
CL	17.0	-6.1168956619	-3.9060269212	1.6383183801
CL	17.0	-6.6931555767	-2.9725258749	-1.0735660224
CL	17.0	-5.1977121185	-5.4273601905	-0.6876783590
CL	17.0	-0.6795659703	-5.3360900350	0.2145135498
CL	17.0	-0.5412884038	-3.6165980235	2.5770953008
CL	17.0	0.7445170051	-2.8137343253	0.0728509391
C	6.0	-3.1163088682	0.4546658490	-0.0992032505
C	6.0	-4.3219260520	1.1369979220	-0.3418519973
C	6.0	-4.3464977719	2.5097242438	-0.4376412246
C	6.0	-3.1614845688	3.2492078422	-0.2945142819
C	6.0	-1.9526052643	2.5827393961	-0.0597279518
C	6.0	-1.9399697927	1.2019220055	0.0365213354
O	8.0	-3.2858754317	4.5845564726	-0.3965948160
C	6.0	-2.1269238550	5.4112762110	-0.2507034673

H	1.0	-5.2365757079	0.5728532990	-0.4500620163
H	1.0	-5.2722302148	3.0384475060	-0.6215313082
H	1.0	-1.0290798142	3.1305361918	0.0481905768
H	1.0	-1.0066049508	0.6899743159	0.2201613643
H	1.0	-2.4803161574	6.4346587377	-0.3482344452
H	1.0	-1.6707404937	5.2810622297	0.7320555776
H	1.0	-1.3949151671	5.2065567558	-1.0342520662

Calculated IR frequencies of 2-(4'-methoxyphenyl)-4,6-bis(trichloromethyl)-1,3,5-triazine (TA)

MODE	FREQ(CM-1)	SYMMETRY	RED.MASS	IR-INTENS.
1	6.092	A	8.506642	0.005359
2	0.256	A	14.594415	0.000017
3	0.220	A	14.398388	0.000007
4	0.132	A	14.546540	0.000005
5	4.155	A	13.425066	0.003395
6	6.380	A	20.804044	0.002416
7	8.128	A	28.081294	0.000168
8	10.609	A	33.042080	0.000688
9	37.215	A	6.393367	0.007371
10	44.358	A	3.720589	0.021022
11	53.605	A	8.925706	0.008749
12	73.005	A	10.041629	0.008957
13	84.267	A	14.275989	0.003262
14	103.070	A	12.044485	0.011052
15	108.039	A	5.149697	0.031229
16	162.061	A	5.913439	0.021938
17	163.496	A	8.879519	0.016461
18	172.311	A	16.832664	0.014811
19	183.501	A	11.689808	0.010612
20	196.574	A	10.409842	0.001579
21	221.098	A	15.144598	0.022358
22	225.977	A	2.619729	0.002186
23	235.287	A	2.038560	0.011268
24	264.489	A	6.579556	0.017340
25	268.054	A	24.063516	0.000762
26	285.755	A	9.053452	0.025777
27	290.662	A	22.631232	0.001648
28	301.055	A	10.855245	0.000874
29	344.150	A	7.575400	0.079375
30	360.968	A	14.173211	0.046780
31	372.993	A	11.647504	0.090303
32	412.862	A	23.250152	0.022674
33	429.013	A	3.051121	0.000188
34	431.053	A	17.088230	0.024361
35	486.850	A	4.750002	0.066165
36	526.455	A	2.680949	0.394532

37	549.254	A	8.478188	0.171773
38	604.962	A	6.351794	1.469085
39	636.190	A	13.317218	0.037568
40	645.936	A	7.190564	0.126996
41	672.918	A	7.141379	1.941389
42	708.207	A	13.054775	2.454218
43	736.642	A	4.888665	2.287486
44	748.299	A	8.553153	2.602260
45	791.716	A	5.915760	2.459348
46	799.180	A	11.964951	0.330920
47	802.254	A	10.934840	2.368592
48	820.823	A	6.586027	0.214901
49	824.437	A	12.326703	1.062632
50	836.082	A	1.280896	0.018162
51	860.653	A	3.470121	0.295590
52	883.390	A	2.761859	0.370519
53	927.312	A	9.659187	0.797791
54	1000.211	A	1.375409	0.006569
55	1011.016	A	1.593450	0.260891
56	1011.880	A	4.714446	1.134296
57	1019.400	A	12.093378	0.383676
58	1021.298	A	2.804890	0.030119
59	1033.375	A	7.657611	1.830434
60	1141.378	A	1.330807	0.246376
61	1153.420	A	3.594054	1.030856
62	1161.359	A	1.275765	0.028037
63	1196.787	A	5.034592	0.685037
64	1200.261	A	1.360549	0.599220
65	1202.439	A	1.479849	3.586653
66	1298.938	A	4.571299	9.034142
67	1325.897	A	2.004818	1.357346
68	1337.135	A	2.691091	3.826989
69	1351.915	A	11.526264	2.496184
70	1390.789	A	9.209121	1.669896
71	1427.607	A	4.513341	15.832303
72	1458.743	A	3.440268	1.200251
73	1472.170	A	1.243220	0.227125
74	1498.151	A	1.053325	0.244932
75	1502.821	A	1.063160	0.786547
76	1534.827	A	9.903341	6.963679
77	1552.563	A	2.977071	0.269821
78	1592.236	A	10.133098	26.266429
79	1601.407	A	6.821858	0.896043
80	1649.818	A	6.094598	2.471316
81	3023.102	A	1.032405	1.301220
82	3090.022	A	1.106940	0.588713
83	3136.064	A	1.101400	0.407323

84	3189.009	A	1.089504	0.072395
85	3204.835	A	1.087849	0.022376
86	3213.486	A	1.092953	0.054387
87	3219.745	A	1.093153	0.197138

Molecular geometry for energy calculation of 2-(4'-methoxyphenyl)-4-(trichloromethyl)-6-(dichloromethyl)-1,3,5-triazine (TA)

N	7.0	-4.2456334460	-1.6804640728	-0.1374739666
C	6.0	-4.1697603114	-2.9797721468	0.0334018400
N	7.0	-3.0731435436	-3.6703306392	0.3224139424
C	6.0	-1.9839841861	-2.9195933742	0.4180878415
N	7.0	-1.9295759718	-1.6133468114	0.2915154262
C	6.0	-3.0950041538	-0.9973352204	0.0221491905
C	6.0	-5.4811533698	-3.7848156211	-0.0492272805
C	6.0	-0.6711061810	-3.6434117218	0.7752851895
CL	17.0	-6.1168956619	-3.9060269212	1.6383183801
CL	17.0	-6.6931555767	-2.9725258749	-1.0735660224
CL	17.0	-5.1977121185	-5.4273601905	-0.6876783590
CL	17.0	-0.6795659703	-5.3360900350	0.2145135498
CL	17.0	0.7445170051	-2.8137343253	0.0728509391
C	6.0	-3.1163088682	0.4546658490	-0.0992032505
C	6.0	-4.3219260520	1.1369979220	-0.3418519973
C	6.0	-4.3464977719	2.5097242438	-0.4376412246
C	6.0	-3.1614845688	3.2492078422	-0.2945142819
C	6.0	-1.9526052643	2.5827393961	-0.0597279518
C	6.0	-1.9399697927	1.2019220055	0.0365213354
O	8.0	-3.2858754317	4.5845564726	-0.3965948160
C	6.0	-2.1269238550	5.4112762110	-0.2507034673
H	1.0	-5.2365757079	0.5728532990	-0.4500620163
H	1.0	-5.2722302148	3.0384475060	-0.6215313082
H	1.0	-1.0290798142	3.1305361918	0.0481905768
H	1.0	-1.0066049508	0.6899743159	0.2201613643
H	1.0	-2.4803161574	6.4346587377	-0.3482344452
H	1.0	-1.6707404937	5.2810622297	0.7320555776
H	1.0	-1.3949151671	5.2065567558	-1.0342520662

4-Fluorotrichlorobenzene

Minimized molecular geometry of 4-fluorotrichlorobenzene

C	6.0	5.5217064763	-8.9998191971	5.4390955842
C	6.0	5.7195605997	-7.6332625242	5.5299360715
C	6.0	6.0780590759	-6.8860587322	4.4013651086
C	6.0	6.2329456145	-7.5328704897	3.1781326734
C	6.0	6.0374960104	-8.9063470072	3.0729848426
C	6.0	5.6863868091	-9.6098480037	4.2060617446
C	6.0	6.2701609461	-5.3945314890	4.5615488137

CL	17.0	6.7862412383	-4.5615490022	3.0588814470
CL	17.0	7.5260936529	-5.0477510295	5.8258955637
CL	17.0	4.7203000162	-4.6161303355	5.1048468929
F	9.0	5.4976966065	-10.9457723356	4.1155545599
H	1.0	5.2490850156	-9.5863012910	6.3054483257
H	1.0	5.5918525015	-7.1502339785	6.4884245850
H	1.0	6.5035963756	-6.9781312879	2.2941477536
H	1.0	6.1584720614	-9.4154692966	2.1268210336

Calculated IR frequencies of 4-fluorotrichlorobenzene

MODE	FREQ (CM^{-1})	SYMMETRY	RED.-MASS	IR-INTENS.
1	0.121	A	14.129935	0.000005
2	0.060	A	14.132532	0.000000
3	0.051	A	14.135112	0.000000
4	7.429	A	15.788030	0.000429
5	11.143	A	12.901268	0.000657
6	14.996	A	29.995824	0.000395
7	41.794	A	3.920800	0.000948
8	83.045	A	13.330007	0.008182
9	153.445	A	16.448099	0.014036
10	195.754	A	12.677340	0.026503
11	234.111	A	14.639163	0.032765
12	243.588	A	16.049013	0.010481
13	268.429	A	20.010641	0.023741
14	332.867	A	15.615227	0.189257
15	388.936	A	7.314933	0.088205
16	390.792	A	27.923634	0.002729
17	428.096	A	3.095175	0.002442
18	433.955	A	5.262011	0.008354
19	538.374	A	2.795228	0.008859
20	563.767	A	9.933239	1.135019
21	647.705	A	7.375820	0.154148
22	687.174	A	5.353110	3.293787
23	758.567	A	4.499816	1.608925
24	771.185	A	10.360561	2.612146
25	813.504	A	7.361408	0.167971
26	833.736	A	1.244343	0.011153
27	871.360	A	1.719030	0.538411
28	886.847	A	7.231825	1.566180
29	976.518	A	1.354620	0.027106
30	992.449	A	1.349556	0.000370
31	1037.814	A	2.589539	0.103451
32	1139.236	A	1.290495	0.051292
33	1188.782	A	1.333660	2.038203
34	1199.544	A	4.124721	0.929261
35	1255.402	A	3.164673	2.270271
36	1330.660	A	4.094191	0.162569

37	1336.534	A	1.610162	0.075217
38	1444.370	A	3.167461	0.195515
39	1544.161	A	2.407651	1.945249
40	1635.907	A	6.863408	0.242441
41	1646.474	A	5.813267	1.603593
42	3194.941	A	1.088728	0.041180
43	3200.169	A	1.091305	0.022229
44	3207.920	A	1.095183	0.009681
45	3229.938	A	1.091627	0.011653

Molecular geometry for energy calculation of 4-fluorodichlorobenzene radical

C	6.0	5.5217064763	-8.9998191971	5.4390955842
C	6.0	5.7195605997	-7.6332625242	5.5299360715
C	6.0	6.0780590759	-6.8860587322	4.4013651086
C	6.0	6.2329456145	-7.5328704897	3.1781326734
C	6.0	6.0374960104	-8.9063470072	3.0729848426
C	6.0	5.6863868091	-9.6098480037	4.2060617446
C	6.0	6.2701609461	-5.3945314890	4.5615488137
CL	17.0	6.7862412383	-4.5615490022	3.0588814470
CL	17.0	7.5260936529	-5.0477510295	5.8258955637
F	9.0	5.4976966065	-10.9457723356	4.1155545599
H	1.0	5.2490850156	-9.5863012910	6.3054483257
H	1.0	5.5918525015	-7.1502339785	6.4884245850
H	1.0	6.5035963756	-6.9781312879	2.2941477536
H	1.0	6.1584720614	-9.4154692966	2.1268210336

Preparation of 1,2,4-trifluoro-5-(trichloromethyl) benzene 2

In a 250 mL four-necked round-bottom flask equipped with reflux condenser, dropping funnel, internal thermometer and mechanical stirrer, 20.7 g of aluminum trichloride were suspended in 75 mL of carbon tetrachloride under nitrogen. This suspension was admixed with 10.2 g of 1,2,4-trifluorobenzene in a gradual manner, such that the reaction mixture was refluxing gently. The resultant HCl gas was introduced into aqueous sodium hydroxide solution. On completion of the addition the mixture was subsequently stirred for 10 min and then cooled down to room temperature. The reaction mixture was discharged onto 300 mL of ice-water and the organic phase was separated off. The organic phase was washed once with 100 mL of water, once with 100 mL of 5% $NaHCO_3$ solution and a further time with 100 mL of water. The carbon tetrachloride was distilled off and the desired product (5.6 g) crystallized out of the oil in the form of colorless fine needles.

^{1}H NMR (400 MHz, CDCl3) δ 8.02 – 7.90 (m, 1H), 6.95 – 6.84 (m, 1H).
m.p.: 118°C

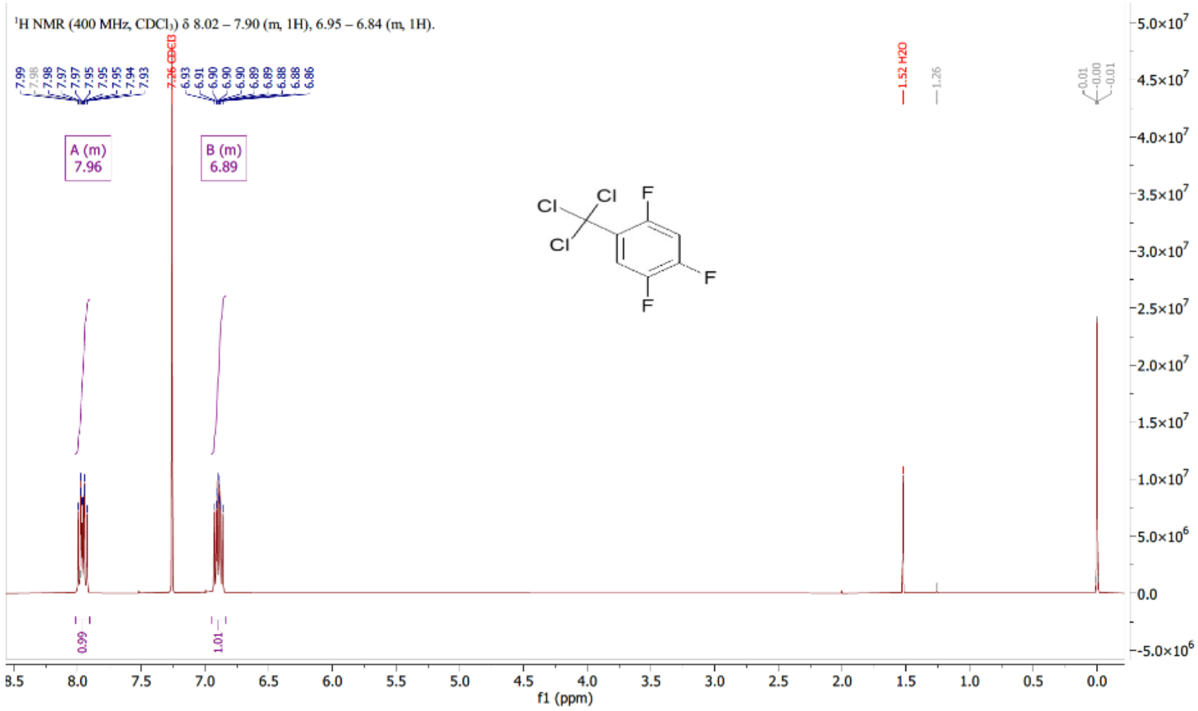

Minimized molecular geometry of 1,2,4-trifluoro-5-(trichloromethyl) benzene 2

C	6.0	-0.6598163040	0.7017966144	0.0483029887
C	6.0	-0.6668474988	-0.6918244016	-0.0230052930
C	6.0	0.5165238788	-1.3983746460	-0.0089928886
C	6.0	1.7331097942	-0.7425307998	0.0780950968
C	6.0	1.7801856032	0.6359888864	0.1485446496
C	6.0	0.5869203079	1.3306992883	0.1312000851
C	6.0	-1.9338013976	1.5176570699	0.0364633752
F	9.0	0.6536546405	2.6715951742	0.1966839611
F	9.0	2.8645963982	-1.4573774245	0.0957561782
F	9.0	0.4997676080	-2.7408743520	-0.0798709980
CL	17.0	-3.4130812134	0.5054248426	-0.0560149368
CL	17.0	-1.9618206741	2.6304154375	-1.3913575058
H	1.0	2.7237078629	1.1583362470	0.2194452725
H	1.0	-1.5910401147	-1.2419357938	-0.0972356258
CL	17.0	-2.0631588912	2.5132038572	1.5439856407

Calculated IR frequencies of 1,2,4-trifluoro-5-(trichloromethyl) benzene 2

MODE	FREQ (CM^{-1})	SYMMETRY	RED.MASS	IR-INTENS.
1	0.107	A	16.523539	0.000002
2	0.037	A	16.532725	0.000000
3	0.029	A	16.523375	0.000000
4	6.156	A	18.415185	0.000239
5	7.569	A	16.976449	0.000297
6	14.448	A	17.107063	0.000034
7	47.270	A	18.198394	0.002640
8	77.439	A	14.339517	0.001489
9	145.349	A	20.720868	0.004257
10	156.835	A	19.620832	0.003447

11	208.098	A	20.107860	0.020603
12	220.350	A	13.361590	0.037291
13	229.885	A	23.202952	0.002600
14	263.205	A	12.008212	0.011212
15	290.317	A	21.076397	0.008049
16	305.117	A	22.180383	0.041457
17	312.914	A	9.778580	0.023584
18	357.765	A	15.903112	0.120110
19	392.905	A	23.830351	0.004030
20	437.966	A	9.011144	0.081197
21	462.445	A	13.411208	0.021374
22	480.964	A	3.422770	0.000102
23	560.147	A	8.805734	0.647318
24	621.172	A	7.363373	0.607828
25	665.545	A	11.336842	1.630445
26	698.924	A	3.880332	0.255976
27	730.971	A	9.522468	1.586360
28	762.203	A	5.743073	1.976318
29	769.474	A	8.106608	1.661931
30	808.106	A	9.653390	1.402292
31	873.884	A	1.634807	0.509664
32	893.094	A	3.903851	0.313284
33	913.758	A	1.440124	0.418350
34	1122.365	A	9.269628	2.549735
35	1173.277	A	1.694830	0.990542
36	1220.197	A	6.375300	1.477096
37	1246.669	A	1.406107	0.143286
38	1328.091	A	11.938120	0.431405
39	1344.661	A	7.911319	2.523572
40	1447.823	A	10.293892	1.498455
41	1535.792	A	4.314932	6.323587
42	1641.731	A	9.559775	0.857651
43	1663.271	A	8.083902	1.591551
44	3213.039	A	1.092996	0.088511
45	3234.944	A	1.090183	0.167757

Molecular geometry for energy calculation of 1,2,4-trifluoro-5-(dichloromethyl) benzene

C	6.0	-0.6598163040	0.7017966144	0.0483029887
C	6.0	-0.6668474988	-0.6918244016	-0.0230052930
C	6.0	0.5165238788	-1.3983746460	-0.0089928886
C	6.0	1.7331097942	-0.7425307998	0.0780950968
C	6.0	1.7801856032	0.6359888864	0.1485446496
C	6.0	0.5869203079	1.3306992883	0.1312000851
C	6.0	-1.9338013976	1.5176570699	0.0364633752
F	9.0	0.6536546405	2.6715951742	0.1966839611
F	9.0	2.8645963982	-1.4573774245	0.0957561782
F	9.0	0.4997676080	-2.7408743520	-0.0798709980

CL	17.0	-3.4130812134	0.5054248426	-0.0560149368
CL	17.0	-1.9618206741	2.6304154375	-1.3913575058
H	1.0	2.7237078629	1.1583362470	0.2194452725
H	1.0	-1.5910401147	-1.2419357938	-0.0972356258
CL	17.0	-2.0631588912	2.5132038572	1.5439856407

Preparation of 1-chloro-4,5-difluoro-2-(trichloromethyl)benzene 3

In a 250 mL four-necked round-bottom flask equipped with reflux condenser, dropping funnel, internal thermometer and mechanical stirrer, 27.4 g of aluminum trichloride were suspended in 100 mL of carbon tetrachloride under nitrogen. This suspension was admixed with 15.3 g of 1-chloro-3,4-difluorobenzene in a gradual manner, such that the reaction mixture was refluxing gently. The resultant HCl gas was introduced into aqueous sodium hydroxide solution. On completion of the addition the mixture was subsequently stirred for 10 min and then cooled down to room temperature. The reaction mixture was discharged onto 300 mL of ice-water and the organic phase was separated off. The organic phase was washed once with 100 mL of water, once with 100 mL of 5% $NaHCO_3$ solution and a further time with 100 mL of water. The carbon tetrachloride was distilled off and little acetonitrile was added. The desired product (6.0 g) crystallized out of this solution in the form of colorless fine needles.
^{1}H NMR (400 MHz, $CDCl_3$) δ 8.26 (dd, J = 11.8, 8.1 Hz, 1H), 7.25 – 7.14 (m, 1H).
m.p.: 137°C

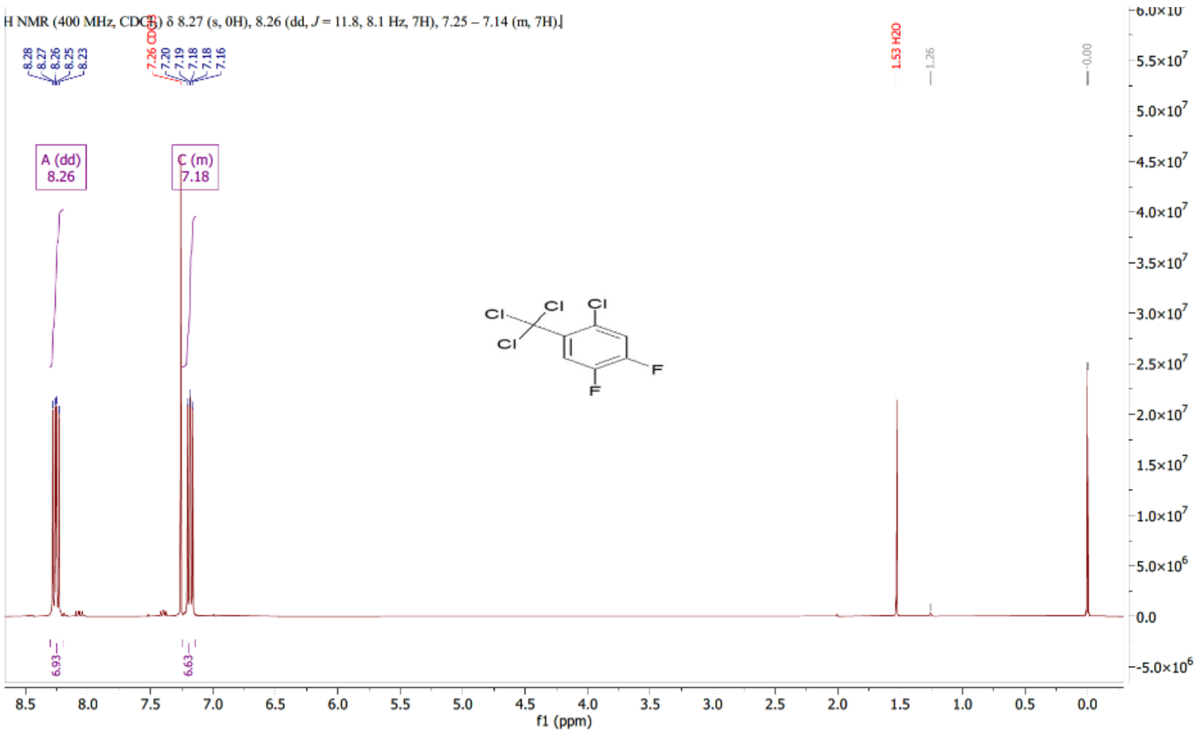

Minimized molecular geometry of 1-chloro-4,5-difluoro-2-(trichloromethyl)benzene 3

C	6.0	-0.6440969274	0.7366044616	0.0498755292
C	6.0	-0.6592113639	-0.6587913998	-0.0155335275
C	6.0	0.5096005871	-1.3864771480	-0.0027987391
C	6.0	1.7327473689	-0.7470695647	0.0748660411

C	6.0	1.7888983476	0.6291040399	0.1407149387
C	6.0	0.6122186062	1.3669346485	0.1291631084
C	6.0	-1.9565808404	1.5069256275	0.0321864319
CL	17.0	0.8135477948	3.0994081507	0.2192846176
F	9.0	2.8588435589	-1.4705417864	0.0891337048
F	9.0	0.4690801606	-2.7269095808	-0.0667891098
CL	17.0	-3.4030120804	0.4389394282	-0.0748144284
CL	17.0	-2.0390081690	2.6106708148	-1.3982682435
H	1.0	2.7493091489	1.1205178664	0.2005390147
H	1.0	-1.5876013720	-1.2006647518	-0.0858335216
CL	17.0	-2.1572242198	2.4761749839	1.5454640637

Calculated IR frequencies of 1-chloro-4,5-difluoro-2-(trichloromethyl)benzene 3

MODE	FREQ (CM^{-1})	SYMMETRY	RED.MASS	IR-INTENS.
1	0.305	A	17.584685	0.000015
2	0.142	A	17.588211	0.000002
3	0.108	A	17.591800	0.000001
4	6.412	A	18.455359	0.000152
5	6.565	A	18.505651	0.000172
6	11.262	A	20.294330	0.000099
7	50.030	A	25.108722	0.002796
8	71.986	A	12.671222	0.000659
9	138.523	A	21.637932	0.003735
10	149.025	A	26.421890	0.006514
11	199.969	A	23.842821	0.014512
12	208.945	A	12.265066	0.025420
13	213.012	A	27.643655	0.003827
14	257.793	A	10.386737	0.000093
15	267.746	A	20.773967	0.012141
16	295.787	A	19.333977	0.011348
17	304.547	A	11.658400	0.040329
18	324.174	A	18.876171	0.060001
19	377.170	A	19.582584	0.033239
20	400.813	A	25.837265	0.012462
21	425.442	A	10.464706	0.031858
22	473.189	A	3.262719	0.007305
23	532.640	A	8.280816	0.377708
24	613.153	A	8.490430	0.774055
25	654.198	A	12.292762	1.259532
26	664.383	A	8.954516	0.664020
27	699.892	A	4.066503	0.276870
28	757.850	A	6.446050	2.321470
29	761.961	A	9.091095	2.274677
30	801.189	A	9.872140	1.463479
31	854.641	A	6.235400	0.557230
32	885.339	A	1.550300	0.165773
33	919.257	A	1.431192	0.437832

34	1026.738	A	6.155696	0.875047
35	1151.777	A	2.228803	1.731842
36	1207.538	A	4.973060	0.682618
37	1259.373	A	1.360507	0.382054
38	1305.731	A	5.281559	1.285720
39	1314.915	A	7.074188	2.112188
40	1409.332	A	9.066620	1.302556
41	1522.142	A	4.231241	5.820245
42	1632.643	A	9.269506	1.695894
43	1648.019	A	7.605862	0.761770
44	3212.747	A	1.092385	0.074206
45	3240.474	A	1.089578	0.213703

Molecular geometry for energy calculation of 1-chloro-4,5-difluoro-2-(dichloromethyl)benzene radical

C	6.0	-0.6440969274	0.7366044616	0.0498755292
C	6.0	-0.6592113639	-0.6587913998	-0.0155335275
C	6.0	0.5096005871	-1.3864771480	-0.0027987391
C	6.0	1.7327473689	-0.7470695647	0.0748660411
C	6.0	1.7888983476	0.6291040399	0.1407149387
C	6.0	0.6122186062	1.3669346485	0.1291631084
C	6.0	-1.9565808404	1.5069256275	0.0321864319
CL	17.0	0.8135477948	3.0994081507	0.2192846176
F	9.0	2.8588435589	-1.4705417864	0.0891337048
F	9.0	0.4690801606	-2.7269095808	-0.0667891098
CL	17.0	-3.4030120804	0.4389394282	-0.0748144284
H	1.0	2.7493091489	1.1205178664	0.2005390147
H	1.0	-1.5876013720	-1.2006647518	-0.0858335216
CL	17.0	-2.1572242198	2.4761749839	1.5454640637

Preparation of 1-bromo-4,5-difluoro-2-(trichloromethyl)benzene 4

In a 250 mL four-necked round-bottom flask equipped with reflux condenser, dropping funnel, internal thermometer and mechanical stirrer, 27.4 g of aluminum trichloride were suspended in 100 mL of carbon tetrachloride under nitrogen. This suspension was admixed with 19.8 g of 1-bromo-3,4-difluorobenzene in a gradual manner, such that the reaction mixture was refluxing gently. The resultant HCl gas was introduced into aqueous sodium hydroxide solution. On completion of the addition the mixture was subsequently stirred for 10 min and then cooled down to room temperature. The reaction mixture was discharged onto 300 mL of ice-water and the organic phase was separated off. The organic phase was washed once with 100 mL of water, once with 100 mL of 5% NaHCO3 solution and a further time with 100 mL of water. The carbon tetrachloride was distilled off and little acetonitrile was added. The desired product (4.4 g) crystallized out of this solution in the form of colourless fine needles.

^{1}H NMR (400 MHz, CDCl3) δ 8.34 (dd, J = 12.1, 8.0 Hz, 1H), 7.41 (dd, J = 9.2, 7.5 Hz, 1H).
m.p.: 135°C

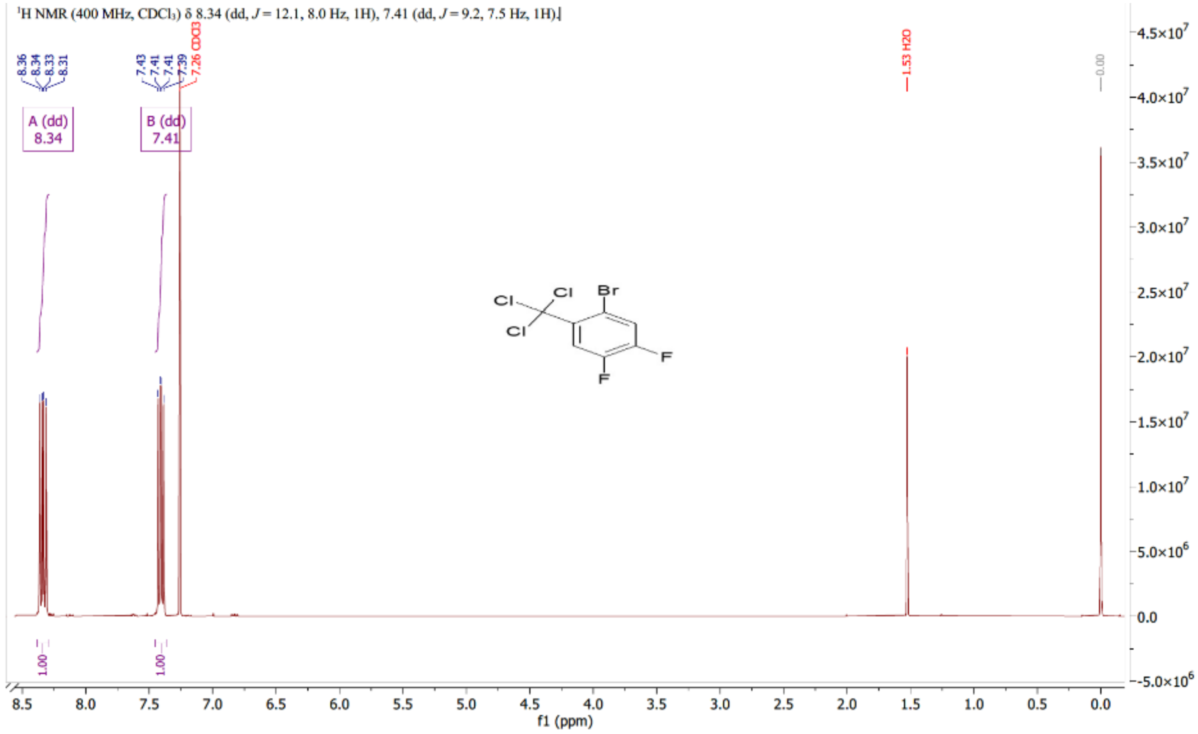

Minimized molecular geometry of 1-bromo-4,5-difluoro-2-(trichloromethyl)benzene 4

C	6.0	5.5090586955	-9.0538532085	5.4086162345
C	6.0	5.6945574169	-7.6787082723	5.5386134874
C	6.0	6.0651948984	-6.8813874747	4.4327966337
C	6.0	6.2346491106	-7.5290570757	3.2016949500
C	6.0	6.0484723882	-8.8924488428	3.0761748499
C	6.0	5.6883477297	-9.6558290731	4.1749436402
C	6.0	6.2860082761	-5.3717467551	4.5211609845
CL	17.0	6.7627419667	-4.6331071923	2.9502193766
CL	17.0	7.6142268509	-4.9809639517	5.6799181727
CL	17.0	4.7752674986	-4.5265088778	5.0341357260
F	9.0	5.5190134035	-10.9796494620	4.0376903229
H	1.0	5.2303157711	-9.6646449606	6.2586597960
BR	35.0	5.4027088823	-7.0069170888	7.3101123541
H	1.0	6.5102453908	-6.9780973132	2.3140448936
F	9.0	6.2203917208	-9.4858804515	1.8819185780

Calculated IR frequencies of 1-bromo-4,5-difluoro-2-(trichloromethyl)benzene 4

MODE	FREQ(CM^{-1})	SYMMETRY	RED.MASS	IR-INTENS.
1	0.069	A	20.538839	0.000000
2	0.057	A	20.532727	0.000000
3	0.043	A	20.516871	0.000000
4	6.074	A	19.798685	0.000080
5	6.522	A	20.048751	0.000108
6	8.963	A	24.235545	0.000091
7	49.485	A	30.668805	0.002692
8	68.199	A	11.620253	0.000234
9	124.142	A	21.209641	0.003619

10	127.550	A	36.598812	0.003195
11	182.234	A	29.794406	0.012515
12	189.545	A	23.762299	0.005780
13	205.747	A	11.890292	0.021203
14	256.522	A	9.717761	0.002097
15	261.711	A	19.309007	0.009268
16	272.408	A	24.036046	0.000491
17	299.714	A	17.365476	0.008815
18	302.902	A	13.176436	0.046328
19	333.826	A	17.584155	0.103837
20	397.780	A	28.284970	0.003320
21	416.931	A	10.759398	0.019864
22	468.224	A	3.232762	0.013915
23	517.321	A	8.294286	0.309148
24	607.894	A	8.693139	0.691179
25	636.067	A	8.896851	0.541520
26	645.474	A	12.441113	1.049758
27	703.808	A	3.735905	0.276460
28	756.061	A	6.545271	2.344221
29	758.394	A	9.259975	2.108481
30	795.154	A	10.193275	1.655251
31	836.031	A	7.173680	0.719125
32	899.015	A	1.564614	0.194531
33	926.207	A	1.460970	0.323109
34	1000.713	A	5.293174	0.511003
35	1148.456	A	2.710319	2.271551
36	1186.788	A	4.071950	0.404671
37	1259.739	A	1.322076	0.390254
38	1297.908	A	3.939552	2.788777
39	1323.871	A	10.837189	0.912916
40	1400.315	A	8.843441	1.335109
41	1520.614	A	4.227877	5.787432
42	1635.169	A	8.868113	1.893191
43	1650.598	A	7.625075	0.718071
44	3214.572	A	1.092811	0.086718
45	3238.732	A	1.090283	0.220098

Molecular geometry for energy calculation of 1-bromo-4,5-difluoro-2-(dichloromethyl)benzene radical

C	6.0	5.5090607588	-9.0538536825	5.4086165669
C	6.0	5.6945608150	-7.6787089974	5.5386141563
C	6.0	6.0651969663	-6.8813877775	4.4327971935
C	6.0	6.2346503517	-7.5290572526	3.2016953857
C	6.0	6.0484721532	-8.8924487209	3.0761745141
C	6.0	5.6883466896	-9.6558288364	4.1749431877
C	6.0	6.2860082327	-5.3717466920	4.5211608824
CL	17.0	6.7627457560	-4.6331078228	2.9502203108

CL	17.0	7.6142237496	-4.9809605700	5.6799203299
F	9.0	5.5190107491	-10.9796487306	4.0376900649
H	1.0	5.2303174974	-9.6646450885	6.2586603093
BR	35.0	5.4027085437	-7.0069171681	7.3101122210
H	1.0	6.5102450603	-6.9780972901	2.3140448617
F	9.0	6.2203876926	-9.4858797466	1.8819178261

Preparation of Components for the Photopolymer Formulation

Preparation of Polyol 1

A 1 l flask was initially charged with 0.18 g of tin octoate, 374.8 g of t-caprolactone and 374.8 g of a difunctional polytetrahydrofuran polyether polyol (equivalent weight 500 g/mol OH), which were heated to 120° C. and kept at this temperature until the solids content (proportion of nonvolatile constituents) was 99.5% by weight or higher. Subsequently, the mixture was cooled and the product was obtained as a waxy solid.

GPC (THF, RI detection, Styrene calibration)

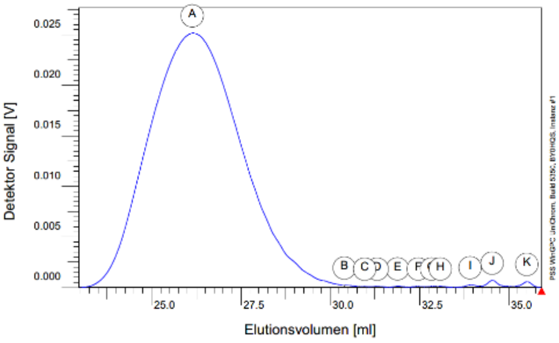

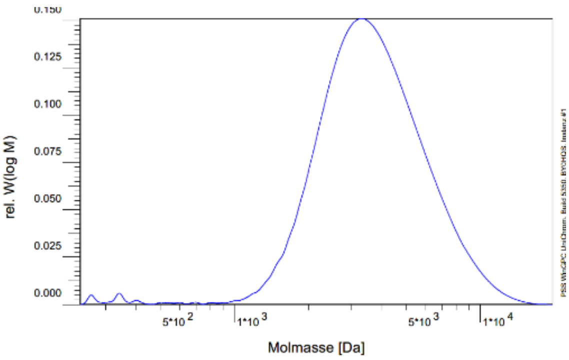

M_n: 3.027 Da; M_w : 4.020 Da

Preparation of Phosphorothoyltrli(oxybenzene-4,1-diylcarbamoyloxyethane-2,1-diyl) trisacrylate

A 500 mL round-bottom flask was initially charged with 0.1 g of 2,6-di-tert-butyl-4-methylphenol, 0.05 g of dibutyltin dilaurate and 213.07 g of a 27% solution of tris(p-isocyanatophenyl) thiophosphate in ethyl acetate (Desmodur® RFE), which were heated to 60° C. Subsequently, 42.37 g of 2-hydroxyethyl acrylate were added dropwise and the mixture was still kept at 60° C. until the isocyanate content had fallen below 0.1%. This was followed by cooling and complete removal of the ethyl acetate under reduced pressure. The product was obtained as a semicrystalline solid.

1H NMR (600 MHz, CDCl3) δ 7.38 (d, J = 8.5 Hz, 6H), 7.21 – 7.11 (m, 6H), 6.96 (s, 3H), 6.45 (dd, J = 17.3, 1.3 Hz, 3H), 6.15 (ddd, J = 17.3, 10.5, 6.8 Hz, 3H), 5.87 (dt, J = 10.5, 2.1 Hz, 3H), 4.41 (s, 12H).

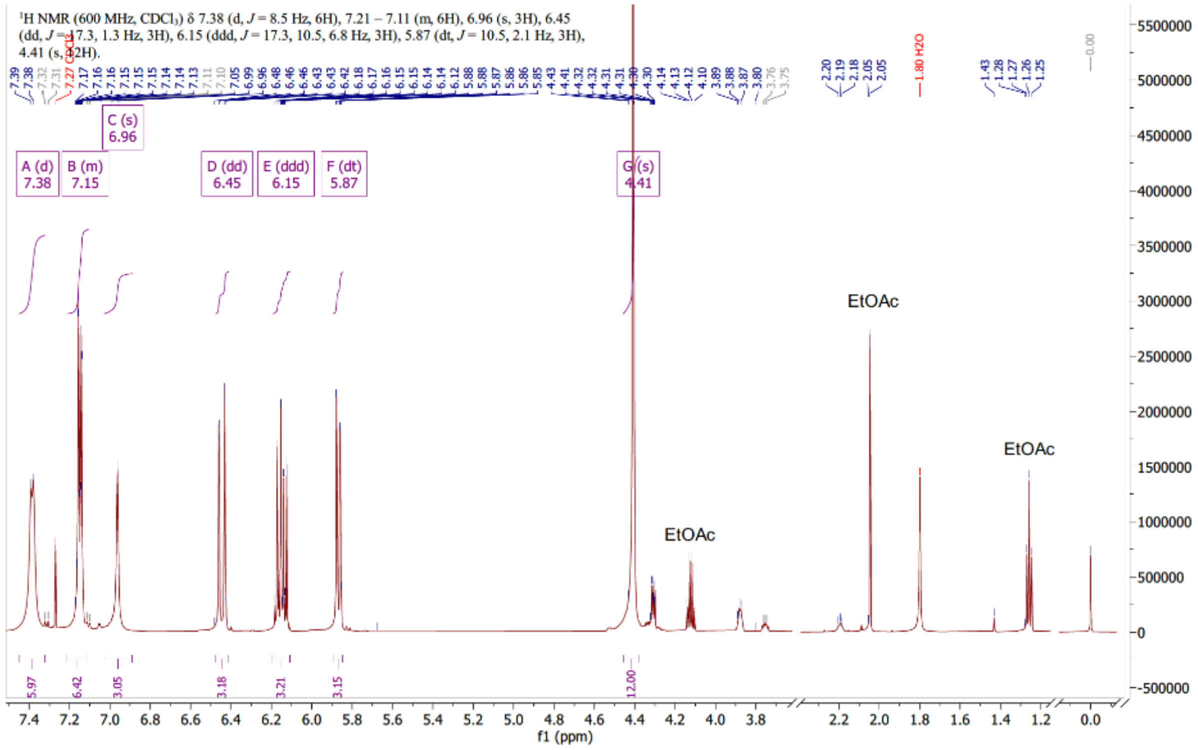

Preparation of 2-(({[3-(Methylsulphanyl)phenyl]-carbamoyl}oxy)ethyl prop-2-enoate

A 100 mL round-bottom flask was initially charged with 0.02 g of 2,6-di-tert-butyl-4-methylphenol, 0.01 g of Desmorapid® Z, 11.7 g of 3-(methylthio)phenyl isocyanate [28479-1-8], and the mixture was heated to 60° C. Subsequently, 8.2 g of 2-hydroxyethyl acrylate were added dropwise and the mixture was still kept at 60° C. until the isocyanate content had fallen below 0.1%. This was followed by cooling. The product was obtained as a colorless liquid.

^1H NMR (600 MHz, Chloroform-d) δ 7.37 (s, 1H), 7.20 (t, J = 7.9 Hz, 1H), 7.12 – 7.06 (m, 1H), 6.96 (ddd, J = 7.8, 1.9, 1.0 Hz, 1H), 6.76 (s, 1H), 6.45 (dd, J = 17.3, 1.3 Hz, 1H), 6.16 (dd, J = 17.4, 10.5 Hz, 1H), 5.87 (dd, J =10.5, 1.4 Hz, 1H), 4.41 (s, 4H), 2.47 (s, 3H).
m.p. 58 °C

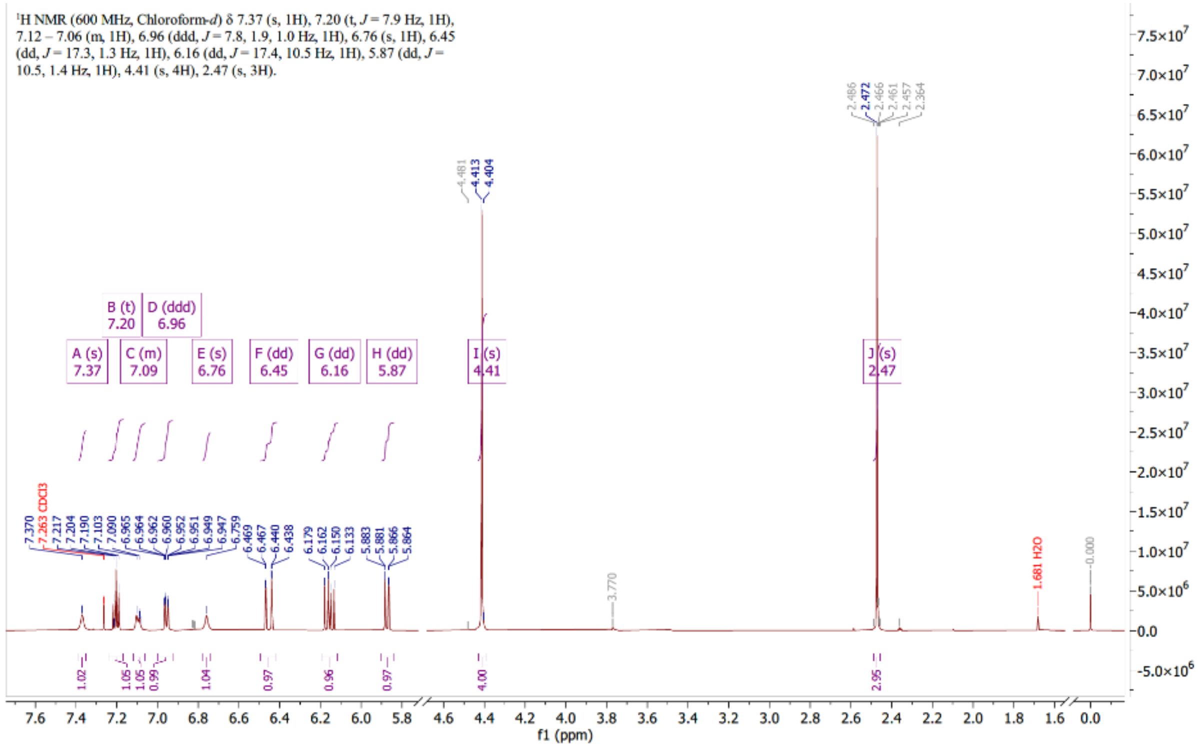

Preparation of bis(2,2,3,3,4,4,5,5,6,6,7,7-dodecafluoroheptyl)(2,2,4-trimethylhexane-1,6-diyl)biscarbamate

A 50 mL round-bottom flask was initially charged with 0.02 g of Desmorapid® Z and 3.6 g of 2,4,4-trimethylhexane 1,6-diisocyanate (TMDI), and the mixture was heated to 60° C. Subsequently, 11.9 g of 2,2,3,3,4,4,5,5,6,6,7,7-dodecafluoroheptan-1-ol were added dropwise and the mixture was still kept at 60° C. until the isocyanate content had fallen below 0.1%. This was followed by cooling. The product was obtained as a colorless oil.

^{1}H NMR (600 MHz, CDCl3) δ 6.05 (s, 2H), 5.43 – 4.86 (m, 2H), 4.59 (qd, J = 14.9, 13.3, 9.6 Hz, 4H), 3.41 – 2.83 (m, 4H), 1.78 – 0.74 (m, 15H).

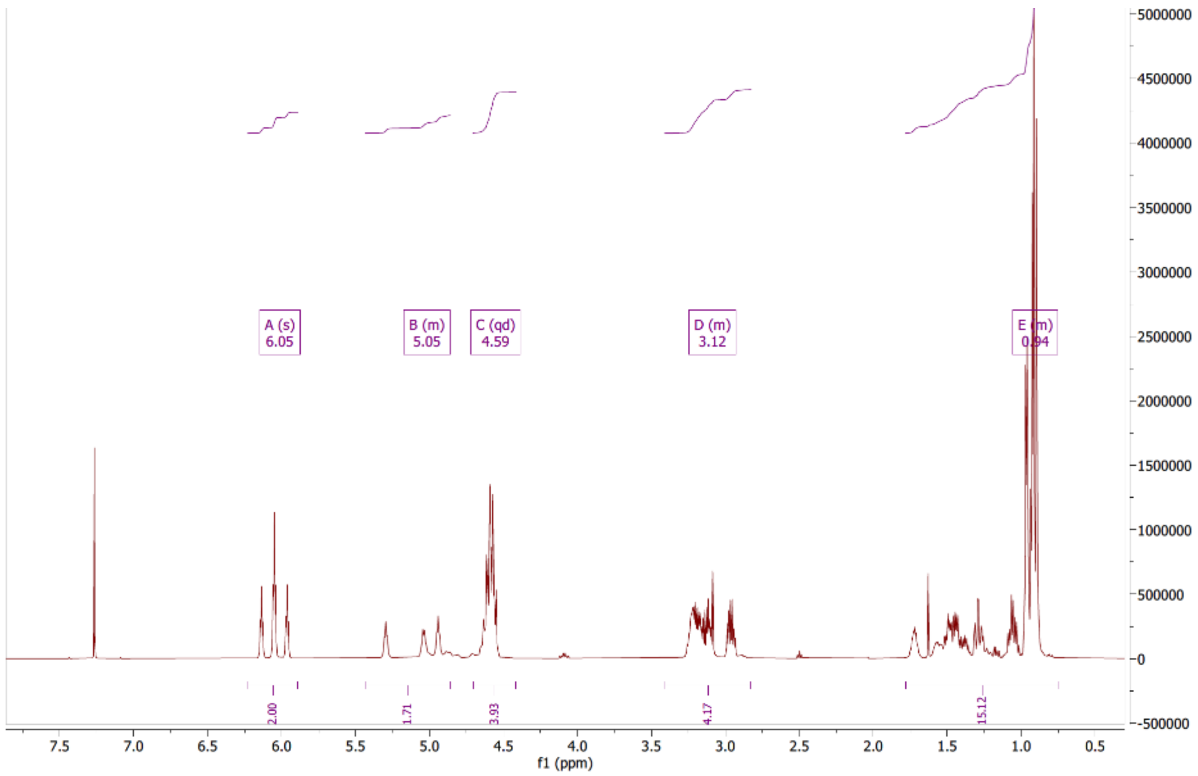

Preparation of borate I: Tetrabutylammonium tris(4-fluorophenyl)(hexyl)borate
A solution of 12.5 g of dibromohexylborane*dimethyl sulphide [64770-04-3] (prepared as described by Brown [2]) in 25 mL of toluene was admixed at 0° C. with 60 mL of a 1.0 M solution of 4-fluorophenylmagnesium bromide [873-77-8] in THF by gradual dropwise addition. The reaction mixture was warmed to room temperature over 30 min and then refluxed for 2 h. Subsequently, the solvent was removed in vacuum and the oily residue was dissolved in 400 mL of methanol/water (4:1) and freed from precipitated solid material by filtration. The solution obtained was admixed with 12.6 g of tetrabutylammonium bromide and the precipitated solid material was isolated by filtration and dried in vacuo. A colorless solid material was obtained in an amount of 10.2 g.

Preparation of borate II: Tetrabutylammonium tris(4-chlorophenyl)(hexyl)borate
A solution of 15.4 g of dibromohexylborane*dimethyl sulphide [64770-04-3] (prepared as described Brown [2]) in 25 mL of toluene was admixed at 0° C with 72 mL of a 0.9 M solution of 4-chlorophenylmagnesium bromide [873-77-8] in THF/toluene by gradual dropwise addition. The reaction mixture was warmed to room temperature over 30 min and then refluxed for 2 h. Subsequently, the solvent was removed in vacuum and the oily residue was dissolved in 400 mL of methanol/water (4:1) and freed from precipitated solid material by filtration. The solution obtained was admixed with 15.6 g of tetrabutylammonium bromide and the precipitated solid material was isolated by filtration and dried in vacuo. A colorless solid material was obtained in an amount of 12.2 g.

Preparation of Safranine O bis(2-ethylhexyl)sulphosuccinate

5.07 g of anhydrous sodium bis(2-ethylhexyl)sulphosuccinate [45297-26-5] were dissolved in 50 mL of anhydrous ethyl acetate. 4.00 g of the anhydrous dye Safranine O [10309-89-4],

composition see [1], were added. The mixture was stirred at room temperature for 3 h and filtered through a fluted filter. The solvent was removed in vacuo to leave 5.1 g of the dye Safranine O bis(2-ethylhexyl)sulphosuccinate [1374689-68-5] as highly viscous oil.

Media to Determine the Holographic Properties

3.38 g of polyol component 1 were mixed with 0.010 g of substance 1, 2.00 g of 2-(({[3-(Methylsulphanyl)phenyl]-carbamoyl}oxy)ethyl prop-2-enoate, 2.00 g of Phosphorothoyltrli-(oxybenzene-4,1-diylcarbamoyloxyethane-2,1-diyl) trisacrylate, 1.50 g of bis(2,2,3,3,4,4,5,5,6,6,7,7-dodecafluoroheptyl)(2,2,4-trimethylhexane-1,6-diyl)biscarbamate, 0.10 g of Tetrabutylammonium tris(4-fluorophenyl)(hexyl)borate, 0.010 g of Safranine O bis(2-ethylhexyl)sulphosuccinate and 0.35 g of N-ethylpyrrolidone at 60° C. to obtain a clear solution. This was followed by cooling to 30° C., admixture of 0.65 g of Desmodur N3900 and renewed mixing. This was finally followed by admixture of 0.01 g of Fomrez UL 28 and renewed brief mixing. The liquid mass obtained was then poured onto a glass plate and covered thereon with a second glass plate. This test specimen was left at room temperature for 12 hours for curing.

Ab initio DFT calculation results of the oxidation potential of tri-chloromethyl compounds, a detailed description on the methodology employed is provided by Nicewicz. [3]

Substance	Triazine S	Triazine A	1	2	3	4
$E_{Radical}$ / a.u.	-4535,132859588	-3462,689943180	-1749,931643771	-1948,476550815	-2308,815946561	-4422,226861354
$E_{Neutral}$ / a.u.	-4535,012744451	-3462,567945447	-1749,809659203	-1948,354208258	-2308,692860079	-4422,095164758
E_{Diff} / Kcal/mol)	-75,4	-76,6	-76,5	-76,8	-77,2	-82,6
E_{ox} / V	-1,15	-1,10	-1,10	-1,09	-1,07	(-0,84)
METHODS EMPLOYED						
DFT functional	B3LYPV1R	B3LYPV1R	B3LYPV1R	B3LYPV1R	B3LYPV1R	B3LYPV1R
Basis set	KTZVPP	KTZVPP	KTZVPP	KTZVPP	KTZVPP	6-31+G(d,p)
Reaction	Isodesmic	Isodesmic	Isodesmic	Isodesmic	Isodesmic	Isodesmic
E(Chloride) / a.u.	-460,3970054	-460,3753284				
DFT functional	B3LYPV1R	B3LYPV1R				
Basis set	KTZVPP	6-31+G(d,p)				

E_{Diff} is calculated by ($E_{Radical}$ – $E_{neutral}$) * 627.5 / kcal/mol.

E_{ox} is calculated by (E_{Diff} / 23.061) – 4.281 – 0.141 / V. [3]

1. Berneth, H.; Rölle, T.; Bruder, F.-K.; Faecke, T.; Weiser, M.-S.; Hoenel, D. Photopolymer formulation for producing holographic media having highly cross-linked matrix polymers. WO2012062655, 2012.
2. Brown, H.C.; Ravindran, N. Direct reaction of dibromoborane-methyl sulfide, HBBr2.cntdot.S(CH3)2, with alkenes. The remarkable reactivity of dibromoborane-methyl sulfide as a hydroborating agent as compared with related dichloroborane derivatives. *Journal of the American Chemical Society* **1977**, *99*, 7097-7098, doi:10.1021/ja00463a071.
3. Nicewicz, D.; Roth, H.; Romero, N. Experimental and Calculated Electrochemical Potentials of Common Organic Molecules for Applications to Single-Electron Redox Chemistry. *Synlett* **2015**, *27*, 714-723, doi:10.1055/s-0035-1561297.
